# Supplementary material for: The mitochondrial Hsp70 controls the assembly of the F1FO-ATP synthase
Source: Nat Commun. 2023 Jan 3;14:39. doi: 10.1038/s41467-022-35720-5 (PMC9810599; doi:10.1038/s41467-022-35720-5)
Supplement: Supplementary file 1 — Supplementary Information [file 41467_2022_35720_MOESM1_ESM.pdf]

SUPPLEMENTARY INFORMATION

The mitochondrial Hsp70 controls the assembly of the F_1F_O-ATP synthase

Jiyao Song, Liesa Steidle, Isabelle Steymans, Jasjot Singh, Anne Sanner, Lena Böttinger, Dominic Winter and Thomas Becker

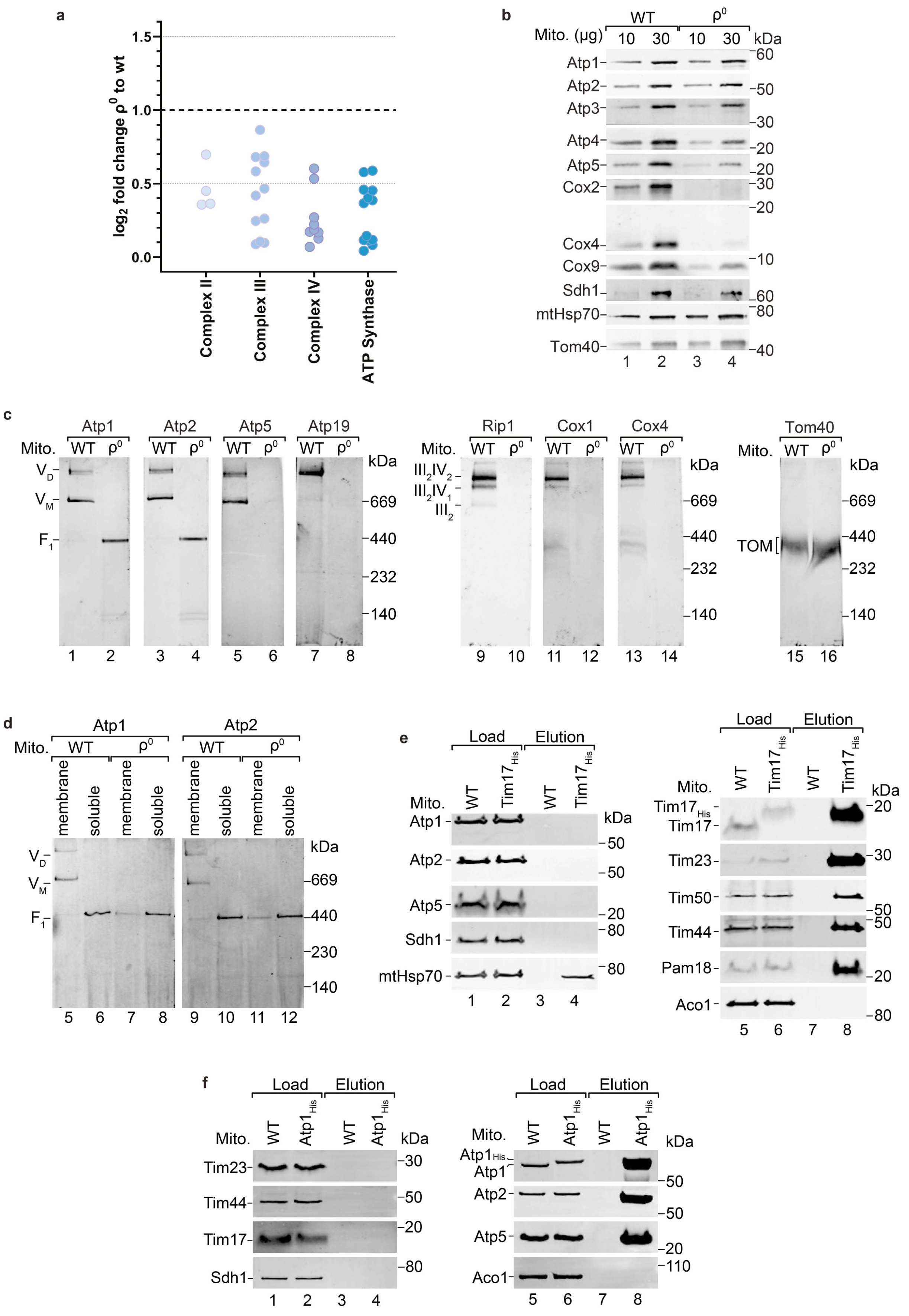
a
log2 fold change ρ0 to wt
1.5
1.0
0.5
0.0
Complex II
Complex III
Complex IV
ATP Synthase
b
WT
ρ0
Mito. (μg)
10 30 10 30
kDa
Atp1
Atp2
Atp3
Atp4
Atp5
Cox2
Cox4
Cox9
Sdh1
mtHsp70
Tom40
1 2 3 4
c
Atp1
Atp2
Atp5
Atp19
Rip1
Cox1
Cox4
Tom40
Mito.
WT ρ0
VD
VM
F1
III2IV2
III2IV1
III2
TOM
669
440
232
140
1 2 3 4 5 6 7 8 9 10 11 12 13 14 15 16
d
Atp1
Atp2
membrane
soluble
669
440
230
140
5 6 7 8 9 10 11 12
e
Load
Elution
Tim17His
Atp1
Atp2
Atp5
Sdh1
mtHsp70
1 2 3 4
Tim17
Tim23
Tim50
Tim44
Pam18
Aco1
5 6 7 8
f
Atp1His
Tim23
Tim44
Tim17
Sdh1
Atp2
Atp5
Aco1
110
1 2 3 4 5 6 7 8

Figure S1 | Characterisation of ρ^0 mitochondria and mtHsp70 populations. (a) Relative protein abundances of components of the respiratory chain complexes and the ATP synthase in ρ^0 mitochondria compared to wild-type (WT) mitochondria. Depicted are log_2 values of fold changes of subunits of complex II (succinate dehydrogenase), complex III (cytochrome bc_1 complex), complex IV (cytochrome *c* oxidase) and ATP synthase. **(b)** The indicated amounts of WT and ρ^0 mitochondria were analysed by SDS-PAGE and immunodetection with the indicated antisera. **(c)** WT and ρ^0 mitochondria were analysed by blue native electrophoresis and immunodetection with the indicated antisera. V_D, dimer of the ATP synthase, V_M, monomer of the ATP synthase, F_1, F_1 domain, IV, complex IV, III, complex III. **(d)** Membrane and soluble fractions of WT or ρ^0 mitochondria were analysed via blue native electrophoresis and immunodetection with the indicated antisera. **(e)** WT and $Tim17_{His}$ mitochondria were subjected to affinity purification via Ni-NTA agarose. Proteins were analysed by SDS-PAGE and immunodetection with the indicated antisera. Load: 1% (left panel) and 4% (right panel), elution: 100%. **(f)** WT and $Atp1_{His}$ mitochondria were subjected to affinity purification via Ni-NTA agarose. Proteins were analysed by SDS-PAGE and immunodetection with the indicated antisera. Load: 1% (right panel) and 4% (left panel), elution: 100%.

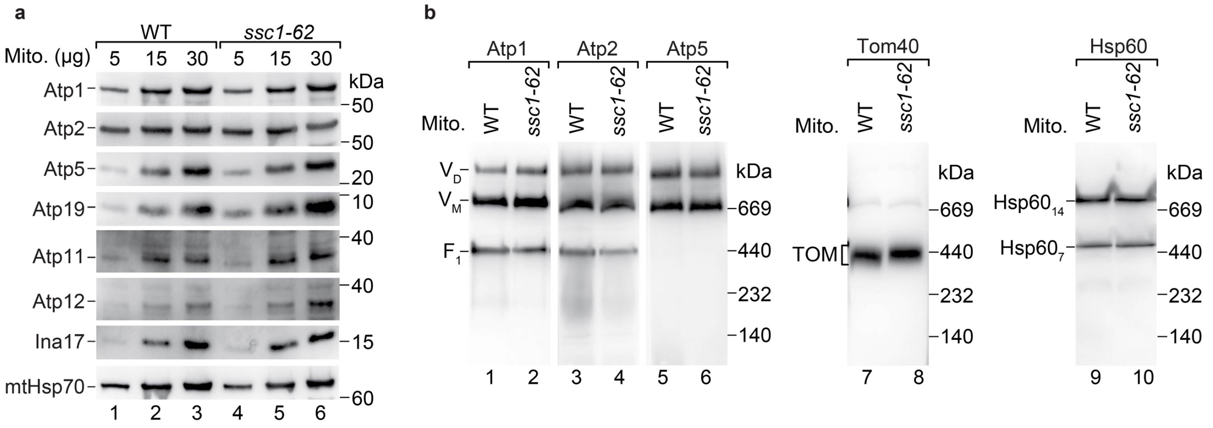

Figure S2 | **Protein levels of *ssc1-62* mitochondria. (a)** The indicated amounts of wild-type (WT) and *ssc1-62* mitochondria were analysed by SDS-PAGE and immunodetection with the indicated antisera. **(b)** WT and *ssc1-62* mitochondria were analysed by blue native electrophoresis and immunodetection with the indicated antisera. V_D, dimer of the ATP synthase, V_M, monomer of the ATP synthase, F_1, F_1 domain.

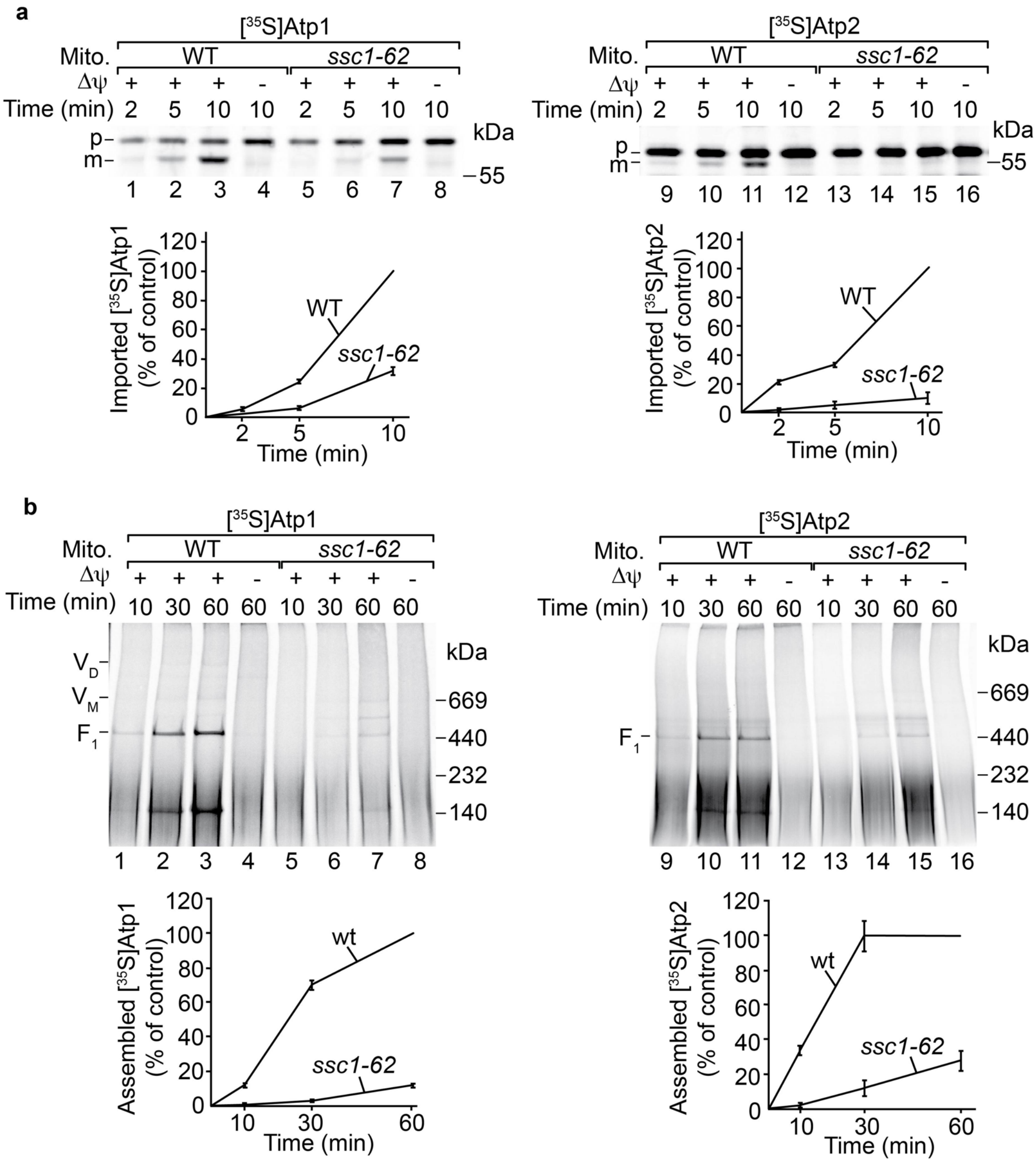

Figure S3 | Import and assembly of Atp1 and Atp2 in *ssc1-62* mitochondria after in vivo heat shock. (a) Upper panels, [35S]Atp1 or [35S]Atp2 were imported for the indicated time points into wild-type (WT) and *ssc1-62* mitochondria isolated from cells grown under non-permissive conditions. In control reactions, the membrane potential ($\Delta\psi$) was depleted. Samples were analysed via SDS-PAGE and autoradiography. p, precursor, m, mature band. Lower panels: Quantification of the imported Atp1 or Atp2. Depicted are mean values ± SEM of 3 independent experiments. The amounts of mature Atp1 or Atp2 in WT mitochondria in the longest import time point were set to 100% (control). Source data are provided as a Source Data file. p, precursor, m, mature band. **(b)** Upper panels, [35S]Atp1 or [35S]Atp2 were imported for the indicated time points into WT and *ssc1-62* mitochondria isolated from cells grown under non-permissive conditions. In control reactions, the membrane potential ($\Delta\psi$) was depleted.

Samples were analysed via blue native electrophoresis and autoradiography. V_D, dimer of the ATP synthase, V_M, monomer of the ATP synthase, F_1, F_1 domain. Lower panels: Quantification of Atp1 or Atp2 assembled into the F_1 domain. Depicted are mean values ± SEM of 3 independent experiments. The amounts of assembled Atp1 or Atp2 in WT mitochondria at the longest import time point were set to 100% (control). Source data are provided as a Source Data file.

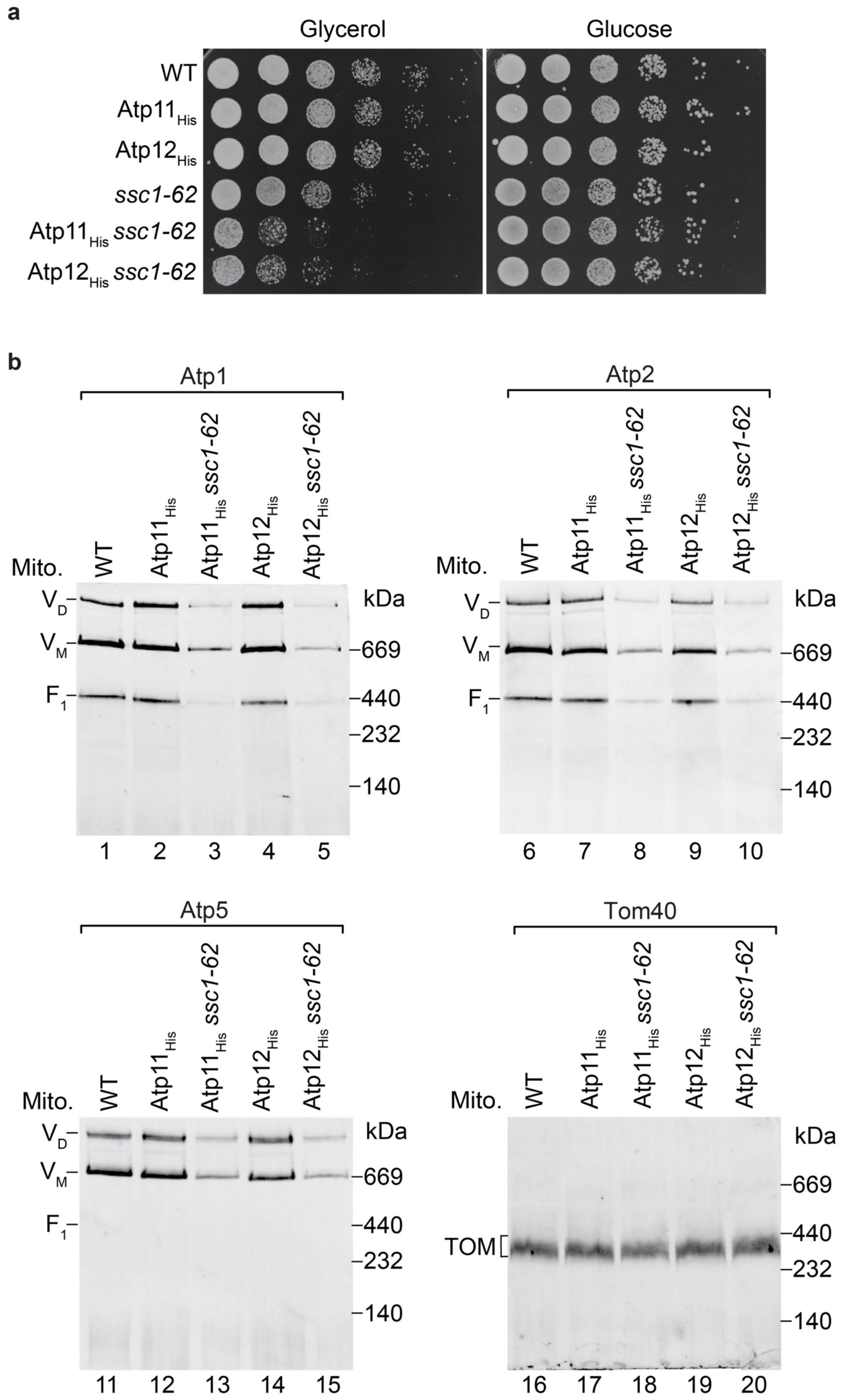

Figure S4 | Functional interaction between mtHsp70 and Atp11 and Atp12. (a) Serial dilutions of the indicated yeast strains were grown at 30°C on full medium containing either glycerol or glucose as carbon source. **(b)** Wild-type (WT), Atp11$_{His}$, Atp11$_{His}$ *ssc1-62*, Atp12$_{His}$ and Atp12$_{His}$ *ssc1-62* mitochondria were analysed by blue native electrophoresis and immunodetection with the indicated antisera. V_D, dimer of the ATP synthase, V_M, monomer of the ATP synthase, F_1, F_1 domain.

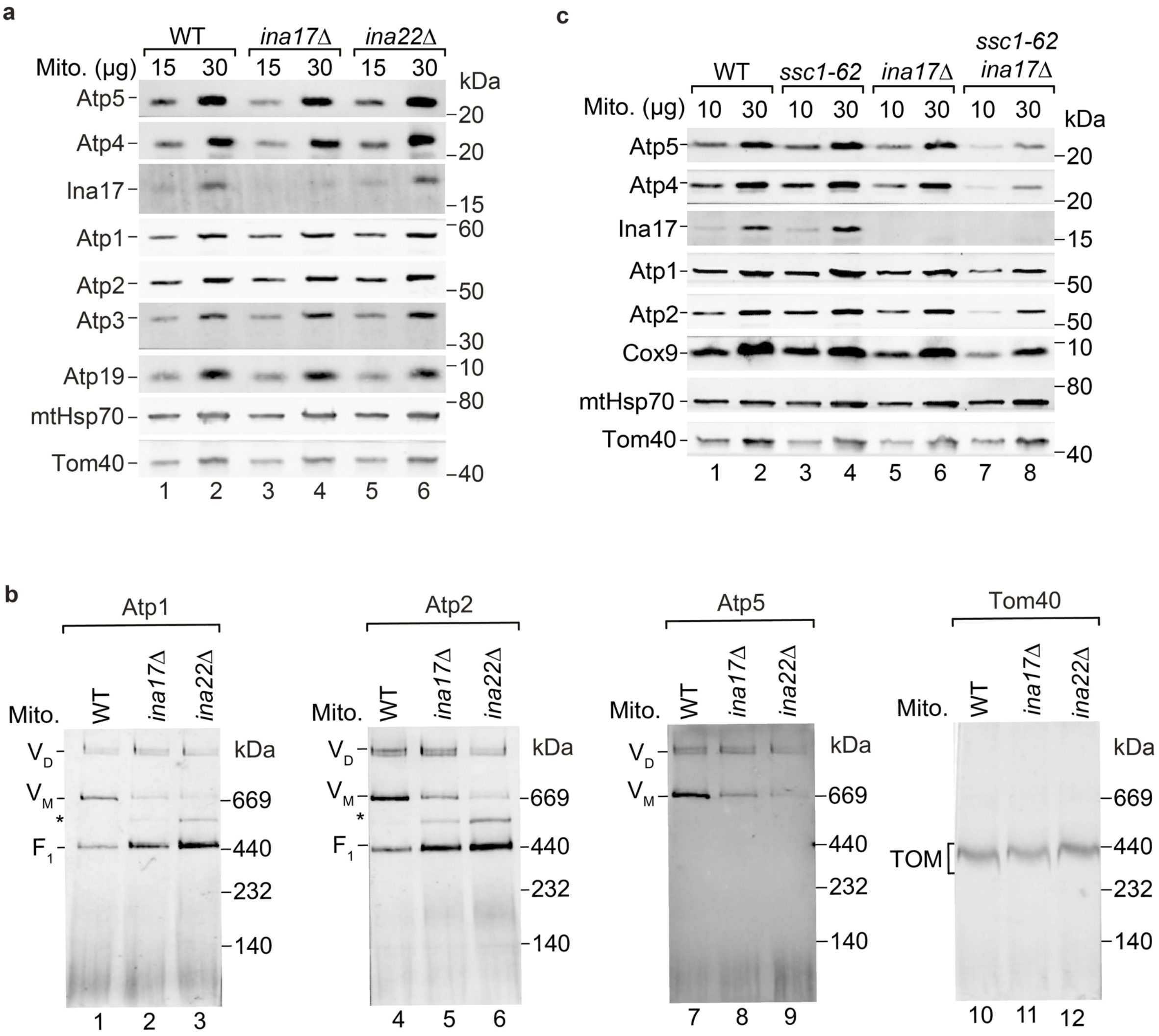

Figure S5 | **Protein levels of *ina17*Δ and *ina22*Δ mitochondria. (a)** The indicated amounts of wild-type (WT), *ina17*Δ and *ina22*Δ mitochondria were analysed by SDS-PAGE and immunodetection with the indicated antisera. **(b)** WT, *ina17*Δ and *ina22*Δ mitochondria were analysed by blue native electrophoresis and immunodetection with the indicated antisera. V_D, dimer of the ATP synthase, V_M, monomer of the ATP synthase, F_1, F_1 domain, asterisk marks unknown Atp1 and Atp2-containing protein complex in *ina17*Δ and *ina22*Δ mitochondria. **(c)** The indicated amounts of WT, *ssc1-62*, *ina17*Δ, *ssc1-62 ina17*Δ mitochondria were analysed by SDS-PAGE and immunodetection with the indicated antisera.

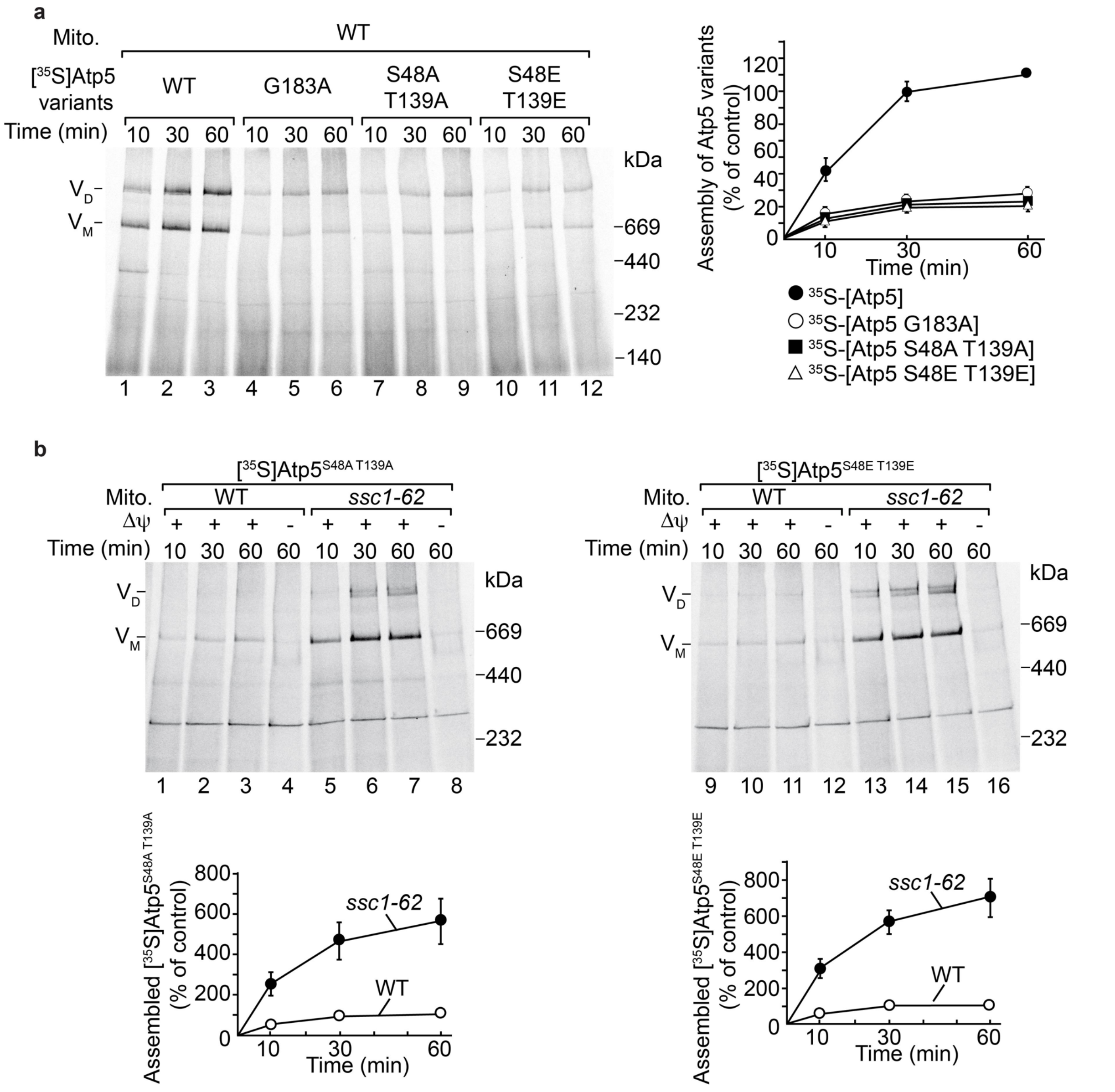

Figure S6 mtHsp70 controls the assembly of Atp5 variants. (a) Left panel, [^{35}S]Atp5, [^{35}S]Atp5^{G183A}, [^{35}S]Atp5$^{S48A\ T139A}$ or [^{35}S]Atp5$^{S48E\ T139E}$ were imported into wild-type (WT) mitochondria for the indicated time points. Samples were analysed via blue native electrophoresis and autoradiography. Right panel, Quantification of the assembly of the Atp5 variants assembled into the monomer and dimer of the ATP synthase. Depicted are mean values ± SEM of 4 independent experiments. The amounts of assembled Atp5 in WT mitochondria at the longest import time point were set to 100% (control). Source data are provided as a Source Data file. **(b)** Upper panels, [^{35}S]Atp5$^{S48A\ T139A}$ or [^{35}S]Atp5$^{S48E\ T139E}$ were imported for the indicated time points into WT and *ssc1-62* mitochondria from cells grown under permissive

conditions. In control reactions, the membrane potential ($\Delta\psi$) was depleted. Samples were analysed via blue native electrophoresis and autoradiography. Lower panels, Quantification of the assembly of [^{35}S]Atp5$^{S48A\ T139A}$ or [^{35}S]Atp5$^{S48E\ T139E}$ into the monomer and dimer of the ATP synthase. Depicted are mean values ± SEM of 4 independent experiments. The amounts of assembled [^{35}S]Atp5$^{S48A\ T139A}$ or [^{35}S]Atp5$^{S48E\ T139E}$ in WT mitochondria at the longest import time point were set to 100% (control). Source data are provided as a Source Data file. V_D, dimer of the ATP synthase, V_M, monomer of the ATP synthase.

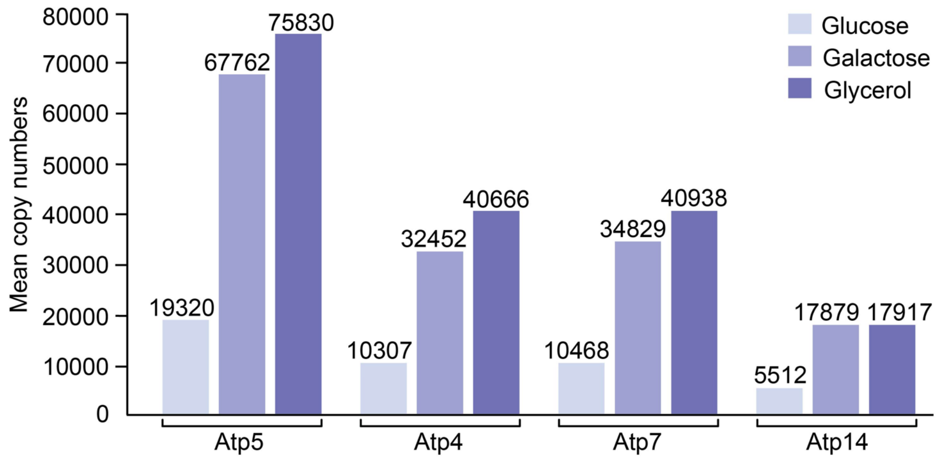

Figure S7 | Protein amounts of peripheral stalk subunits in the cell. Depicted are the copy numbers of the ATP synthase subunits of the peripheral stalk in cells grown in different media as determined previously[1].

SUPPLEMENTAL TABLES

Table S1 I *Saccharomyces cerevisiae* strains used in this study

Strain	Genetic background	Source	No.
YPH499 (WT)	*MATa ura3-52 lys2-801_amber ade2-101_orchre trp1-Δ63 his3-Δ200 leu2-Δ1*	ref. 2	1501
BY4741 (WT)	*MATa his3Δ1 leu2Δ0 met15Δ0 ura3Δ0*	Euroscarf	1354
BY4741 ρ^0	BY4741 ρ^0	Becker lab	1505
mtHsp70$_{His}$	YPH499 *ssc1::SSC1$_{His}$-HIS3MX6*	Ref. 3	2405
YPH499 ρ^0 mtHsp70$_{His}$	YPH499 ρ^0 *ssc1::SSC1$_{His}$-HIS3MX7*	this study	TB220
atp3Δ	BY4741 *atp3::kanMX4*	Euroscarf	TB175
atp4Δ	BY4741 *atp4::kanMX4*	Euroscarf	TB176
atp5Δ	BY4741 *atp5::kanMX4*	Euroscarf	TB177
atp7Δ	BY4741 *atp7::kanMX4*	Euroscarf	TB178
atp14Δ	BY4741 *atp14::kanMX4*	Euroscarf	TB179
ina17Δ	BY4741 *ina17::kanMX4*	Euroscarf	TB180
ina22Δ	BY4741 *ina22::kanMX4*	Euroscarf	TB181
Atp1$_{His}$	YPH499 *atp1::ATP1$_{His}$-HIS3MX6*	this study	5414
Atp11$_{His}$	YPH499 *atp11::ATP11$_{His}$-hphNT1*	this study	5416
Atp12$_{His}$	YPH499 *atp12::ATP12$_{His}$-hphNT1*	this study	5417
Ina17$_{His}$	YPH499 *ina17::INA17$_{His}$-hphNT1*	this study	5418
Ina22$_{His}$	YPH499 *ina22::INA22$_{His}$-hphNT1*	this study	5419
Ssc1 shuffle strain	YPH499 *SSC1::ADE2* pYEp352-Ssc1-WT	Ref. 3	3251
ssc1-WT	YPH499 *SSC1::ADE2* pFL39-*SSC1*	Ref. 3	2535
ssc1-62	YPH499 *SSC1::ADE2* pFL39-*SSC1-62*	Ref. 3	2546
Atp11$_{His}$ *ssc1-62*	YPH499 *SSC1::ADE2* pFL39-*SSC1-62 atp11::ATP11$_{HIS}$-hphNT1*	this study	5424
Atp12$_{His}$ *ssc1-62*	YPH499 *SSC1::ADE2* pFL39-*SSC1-62 atp12::ATP12$_{HIS}$-hphNT1*	this study	5425
ssc1-WT ina17Δ	YPH499 *SSC1::ADE2* pFL39-*SSC1 ina17::HIS3MX6*	this study	5430
ssc1-WT ina22Δ	YPH499 *SSC1::ADE2* pFL39-*SSC1 ina22::HIS3MX6*	this study	5431
ssc1-WT fmc1Δ	YPH499 *SSC1::ADE2* pFL39-*SSC1 fmc1::HIS3MX6*	this study	5440
ina17Δ Ssc1 shuffle strain	YPH499 *SSC1::ADE2* pYEp352-Ssc1-WT *ina17::HIS3MX6*	this study	TB185
Ina22Δ Ssc1 shuffle strain	YPH499 *SSC1::ADE2* pYEp352-Ssc1-WT *ina17::HIS3MX6*	this study	TB188
fmc1Δ Ssc1 shuffle strain	YPH499 *SSC1::ADE2* pYEp352-Ssc1-WT *fmc1::HIS3MX6*	this study	TB189
ssc1-62 ina17Δ	YPH499 *SSC1::ADE2* pFL39-*SSC1-62 ina17::HIS3MX6*	this study	5438

*ssc1-62 ina22*Δ	YPH499 *SSC1::ADE2* pFL39-*SSC1-62 ina22::HIS3MX6*	this study	5439
*ssc1-62 fmc1*Δ	YPH499 *SSC1::ADE2* pFL39-*SSC1-62 fmc1::HIS3MX6*	this study	5442
ssc1-WT + *pRS415*	YPH499 *SSC1::ADE2* pFL39-*SSC1* pRS415	this study	5443
ssc1-WT + *ATP5*↑	YPH499 *SSC1::ADE2* pFL39-*SSC1* pRS415-*GAL1-ATP5*	this study	5444
ssc1-WT + *ATP5*^{G183A}↑	YPH499 *SSC1::ADE2* pFL39-*SSC1* pRS415-*GAL1-ATP5*^{G183A}	this study	TB182
ssc1-62 + *pRS415*	YPH499 *SSC1::ADE2* pFL39-*SSC1-62* pRS415	this study	5447
ssc1-62 + *ATP5*↑	YPH499 *SSC1::ADE2* pFL39-*SSC1-62* pRS415-*GAL1-ATP5*	this study	5448
ssc1-62 + *ATP5*^{G183A}↑	YPH499 *SSC1::ADE2* pFL39-*SSC1-62* pRS415-*GAL1-ATP5*^{G183A}	this study	TB183
ssc1-WT + *ATP5*$_{HA}$↑	YPH499 *SSC1::ADE2* pFL39-*SSC1* pRS415-*GAL1-ATP5*$_{HA}$	this study	TB222
ssc1-WT + *ATP5*$^{G183A}_{HA}$↑	YPH499 *SSC1::ADE2* pFL39-*SSC1* pRS415-*GAL1-ATP5*$^{G183A}_{HA}$	this study	TB223
ssc1-62 + *ATP5*$_{HA}$↑	YPH499 *SSC1::ADE2* pFL39-*SSC1-62* pRS415-*GAL1-ATP5*$_{HA}$	this study	TB224
ssc1-62 + *ATP5*$^{G183A}_{HA}$↑	YPH499 *SSC1::ADE2* pFL39-*SSC1-62* pRS415-*GAL1-ATP5*$^{G183A}_{HA}$	this study	TB225
*pim1*Δ	BY4741 *pim1::kanMX4*	Euroscarf	KO 2990
*yta12*Δ	BY4741 *yta12::kanMX4*	Euroscarf	KO 2462
*pim1*Δ + *ATP5*$_{HA}$↑	BY4741 *pim1::kanMX4* pRS415-*GAL1-ATP5*$_{HA}$	this study	TB226
*pim1*Δ + *ATP5*$^{G183A}_{HA}$↑	BY4741 *pim1::kanMX4* pRS415-*GAL1-ATP5*$^{G183A}_{HA}$	this study	TB227
*yta12*Δ + *ATP5*$_{HA}$↑	BY4741 *yta12::kanMX4* pRS415-*GAL1-ATP5*$_{HA}$	this study	TB230
*yta12*Δ + *ATP5*$^{G183A}_{HA}$↑	BY4741 *yta12::kanMX4* pRS415-GAL1-*ATP5*$^{G183A}_{HA}$	this study	TB231
Tim17$_{His}$	YPH499 *tim17::TIM17-HIS-HIS3MX6*	Ref. 4	5387

Table S2 | Plasmids used in this study

Plasmid	Source	No.
pRS415	Ref. 5	X 24
pRS415-GAL1-ATP5	this study	5138
pRS415- GAL1-ATP5^{G183A}	this study	TB93
pRS415-GAL1-ATP5$_{HA}$	this study	TB130
pRS415- GAL1-ATP5$^{G183A}_{HA}$	this study	TB131
pFA6a-His10-HIS3MX6	Ref. 6	X 63
pFA6a-hphNT1	Ref. 7	2722
pFA6a-HIS3MX6	Ref. 7	1424

Table S3 | Oligonucleotides used in this study

Name	Sequence	Source
Atp1_forw	ATCGATTTAGGTGACACTATAGAATACGCCGCCGCCA TGTTGGCTCGTACTGCTGC	this study
Atp1+2Met_rev	TTA CAT CAT AAA AGT GGC AAC AAA TGA TTC AG	this study
Atp5_forw	ATCGATTTAGGTGACACTATAGAATACGCCGCCGCCA TGTTTAATAGAGTCTTTACC	this study
Atp5+3Met_rev	TTA CAT CAT CAT AAT GCT GTC CTC TAA GAC C	this study
Atp4_forw	ATC GAT TTA GGT GAC ACT ATA GAA TAC GCC GCC GCC ATG AGC ATG AGT ATG GGT GTC	this study
Atp4+2Met_rev	GAT CTT ACA TCA TCT TCA ATT TAG AAA GCA ATT G	this study
Atp11-His fw	GAT TCA CCG TCG AGA AAT TGA TTT CGC TAT CAC AGT CCA TGG AAA ATC GTA CGC TGC AGG TCG AC	this study
Atp11-His rev	CAT TAA TAT ATA TAT ATA TAT ATA TAT ACG TAT ACG GAA GTA ATT CTT CGT TAA TCG ATG AAT TCG AGC TCG	this study
Atp12-His fw	CAT CAG AAG AAA AAT TCA TAC TGC TGC GAT TGC TGC TTT TAA GCA ACG TAC GCT GCA GGT CGA C	this study
Atp12-His rev	GTA TAT ATA TCA TAG AAA AGT AGT AAT AGA ACA ACA ATT GAA TCA ACC ATA TTA ATC GAT GAA TTC GAG CTC G	this study
Ina17-His fw	GTA ATG AAT CAA CAC ATA TGA AGC CTT GGT ATA GGA GAT GGT TTT GGC GTA CGC TGC AGG TCG AC	this study
Ina17-His rev	GGT ACA TTC TAG AAA CAC GAA AAG CCA ACA AGA TAA CTA ATT CTC AAT CGA TGA ATT CGA GCT CG	this study
Ina22-His fw	GAT TTG AAT GAA ATC GCC AAA GAA CAT GAT AAA ATC CCA AAA TTT CTA CGT ACG CTG CAG GTC GAC	this study
Ina22-His rev	CTT GCG TTA TAT TTA CAT GTG GTA TAT CCG GAT GCA TAG AGC CTA CTA ATC GAT GAA TTC GAG CTC G	this study

Ina17 delta fw	CACTCTGTTATATTTGTAAACTTTGAGAAAGGAAATGGGAACCAAAGCATATAATAGACAATCGGATCCCCGGGTTAATTAA	this study
Ina17 delta rev	AAATATTTACATAGGCAATGGTACATTCTAGAAACACGAAAAGCCAACAAGATAACTAATTCGAATTCGAGCTCGTTTAAAC	this study
Ina22 delta fw	ATATAATGAGCAGGGAAAAAGTAACAACAAGGAAAGACAAGTCATACGTAAAAGGTGTAAGGCGGATCCCCGGGTTAATTAA	this study
Ina22 delta rev	TGTAAAATTATTTATTCGCTTGCGTTATATTTACATGTGGTATATCCGGATGCATAGAGCCGAATTCGAGCTCGTTTAAAC	this study
GAL1pro-Atp5 fw	TCAAGGAGGTCGACATGTTTAATAGAGTCTTTACC	this study
Atp5-GAL1pro rv	TTCTAATCCGCTCGAGGGGGGGCCCGGTACCCAAT	this study
Fmc1 delta fw	TTTCATTTGCAGCGATAAGTGGATAACAAAAAGCTGAAAAGAGAGAGAAAGAGAGAGTGCCAGGGAACGGATCCCCGGGTTAATTAA	this study
Fmc1 delta rv	CCTAGATATTTACATCAAATATATATATATACTTATTTACAAAACTCTGATATTATAAATTGATTAGGAATTCGAGCTCGTTTAAAC	this study
Atp5 S48A fw	CAGGCCGCTGCAAAGAACGCTTCTATTGATGCTGCCTTTC	this study
Atp5 S48A rv	GAAAGGCAGCATCAATAGAAGCGTTCTTTGCAGCGGCCTG	this study
Atp5 S48E fw	CAGGCCGCTGCAAAGAACGAATCTATTGATGCTGCCTTTC	this study
Atp5 S48E rv	GAAAGGCAGCATCAATAGATTCGTTCTTTGCAGCGGCCTG	this study
Atp5 T139A fw	CAACGGGTTACTTAAAGGTGCTGTGACTAGTGCTGAACC	this study
Atp5 T139A rv	GGTTCAGCACTAGTCACAGCACCTTTAAGTAACCCGTTG	this study
Atp5 T139E fw	CAACGGGTTACTTAAAGGTGAAGTGACTAGTGCTGAACC	this study
Atp5 T139E rv	GGTTCAGCACTAGTCACTTCACCTTTAAGTAACCCGTTG	this study
Atp5 G183A fw	AGCCAGAGATTAAGGGTGCTTTGATTGTAGAACTTGG	this study
Atp5 G183A rv	CCAAGTTCTACAATCAAAgCACCCTTAATCTCTGGCT	this study
Atp1 His fw	TTGTCTAAAGAATTGTTGGCATCTCTAAAGAGTGCTACTGAATCATTTGTTGCCACTTTTCGTACGCTGCAGGTCGAC	this study
Atp1 His rv	TATTTACATTTCTTTTTGAGACGTACCTTATATTCATTTTTATTTTTTTAGTTCACATTAATCGATGAAATTCGAGCTCG	this study
Ssc1His fw	TAACAACAACAACAACAACAACGGCAACAATGCCGAATCTGGTGAAACTAAGCAGCGTACGCTGCAGGTCGAC	this study

Ssc1His rv	ATTCTTTCTAGTTTAACATTGTGGTAGTAATTTATTAAC AGGAATTTGCTTTTTAATCGATGAATTCGAGCTCG	this study

Table S4 | Antibodies used in this study

Antigen	Dilutiuon	Number / Company	Secondary antibody	Reference
Atp1	1 : 500	GR5075-3	anti-rabbit	Ref. 8
Atp2	1 : 1000	GR863-4	anti-rabbit	Ref. 8
Atp3	1 : 250	GR1671-4	anti-rabbit	Ref. 9
Atp4	1 : 250	GR1970-4	anti-rabbit	Ref. 9
Atp5	1 : 250	GR1546-4	anti-rabbit	Ref. 8
Atp11	1 : 200	GR5152-2	anti-rabbit	this study
Atp12	1 : 200	GR5155-2	anti-rabbit	this study
Atp19	1 : 250	GR1961-3	anti-rabbit	Ref. 9
Atp20	1 : 1000	GR1516-4	anti-rabbit	Ref. 8
Ina17	1 : 500	GR3340-2	anti-rabbit	Ref. 10
mtHsp70	1 : 1000	GR1830-3	anti-rabbit	Ref. 9
Hsp60	1 : 1000	GR170-2	anti-rabbit	Ref. 11
Cox2	1 : 250	GR1948-4	anti-rabbit	Ref. 9
Cox4	1 : 1000	GR578-5	anti-rabbit	Ref. 9
Cox9	1 : 500	GR3611-3	anti-rabbit	Ref. 11
Sdh1	1 : 250	GR1848-5	anti-rabbit	Ref. 11
Rip1	1 : 1500	GR543-4	anti-rabbit	Ref. 11
Mge1	1 : 500	GR1837-2	anti-rabbit	Ref. 11
Mdh1	1 : 1000	GR1089-4	anti-rabbit	Ref. 11
Mss51	1 : 1000	GR1952-5	anti-rabbit	Ref. 11
Tom40	1 : 1000	GR168-5	anti-rabbit	Ref. 12
Tom70	1 : 500	GR657-3	anti-rabbit	Ref. 12
Tim44	1 : 250	GR1835-4	anti-rabbit	Ref. 9
Tim17	1 : 500	GR1844-6	anti-rabbit	Ref. 9
Tim23	1 : 500	GR3878-4	anti-rabbit	Ref. 9
Tim50	1 : 500	GR3881-3	anti-rabbit	Ref. 9
Pam16	1 : 250	GR3121-4	anti-rabbit	Ref. 13
Pam18	1 : 250	GR751-2	anti-rabbit	Ref. 13
Mdj1	1 : 250	GR121-7	anti-rabbit	Ref. 9
Aco1	1 : 1000	GR945-3	anti-rabbit	Ref. 11
Pim1	1 : 250	GR626-5	anti-rabbit	Ref. 14

References

1. Morgenstern, M. *et al.* Definition of a high-confidence mitochondrial proteome at quantitative scale. *Cell Rep.* **19**, 2836-2852 (2017).
2. Janke, C. *et al.* A versatile toolbox for PCR-based tagging of yeast genes: new fluorescent proteins, more markers and promoter substitution cassettes. Yeast **21**, 947-962 (2004).
3. Böttinger, L. *et al.* A complex of Cox4 and mitochondrial Hsp70 plays an important role in the assembly of the cytochrome *c* oxidase. *Mol. Biol. Cell* **24**, 2609-2619 (2013).
4. Priesnitz, C. *et al.* Coupling to Pam16 differentially controls the dual role of Pam18 in protein import and respiratory chain formation. *Cell Rep.* **39,** 110619 (2022).
5. Becker, T. *et al*. The mitochondrial import protein Mim1 promotes biogenesis of multispanning outer membrane proteins. *J. Cell Biol*. **194**, 387–395 (2011).
6. Knop, M. *et al*. Epitope tagging of yeast genes using a PCR-based strategy: more tags and improved practical routines. *Yeast* **15**, 963–972 (1999).
7. Longtine, M. S. *et al*. Additional modules for versatile and economical PCR-based gene deletion and modification in saccharomyces cerevisiae. *Yeast* **14**, 953–961 (1998)
8. Rampelt, H. *et al*. Dual role of Mic10 in mitochondrial cristae organization and ATP synthase-linked metabolic adaptation and respiratory growth. *Cell Rep.* **38**,110290 (2022)
9. Böttinger, L. *et al.* A complex of Cox4 and mitochondrial Hsp70 plays an important role in the assembly of the cytochrome *c* oxidase. *Mol. Biol. Cell* **24**, 2609-2619 (2013).
10. Lytovchenko, O. *et al.* The INA complex facilitates assembly of the peripheral stalk of the mitochondrial F_1F_O-ATP synthase. *EMBO J.* **33**, 1624-38 (2014).
11. Böttinger, L. *et al.* Mitochondrial heat shock protein (Hsp) 70 and Hsp10 cooperate in the formation of Hsp60 complexes. *J. Biol. Chem.* **290**, 11611-11622 (2015).
12. Mårtensson, C.U. *et al.* Mitochondrial protein translocation-associated degradation. *Nature* **569**, 679-683 (2019)
13. Priesnitz, C. *et al.* Coupling to Pam16 differentially controls the dual role of Pam18 in protein import and respiratory chain formation. *Cell Rep.* **39,** 110619 (2022).
14. Bender, T., Leidhold, C., Ruppert, T., Franken, S. & Voos, W. The role of protein quality control in mitochondrial protein homeostasis under oxidative stress. *Proteomics* **10,** 1426-1443 (2010)
