# Supplementary material for: The mitochondrial Hsp70 controls the assembly of the F1FO-ATP synthase
Source: Nat Commun. 2023 Jan 3;14:39. doi: 10.1038/s41467-022-35720-5 (PMC9810599; doi:10.1038/s41467-022-35720-5)
Supplement: Supplementary file 3 — Description of Additional Supplementary Files [file 41467_2022_35720_MOESM3_ESM.pdf]

File name: Supplementary Data 1

Description: List of proteins identified in isolated mitochondria experiments (ρ^0/WT) by quantitative MS.

File name: Supplementary Data 2

Description: List of proteins identified in mtHsp70$_{His}$ pull-down experiments (ρ^0/WT) by quantitative MS.

File name: Supplementary Data 3

Description: List of proteins identified in Mge1$_{His}$ pull-down experiments (ρ^0/WT) by quantitative MS.
