# Supplementary material for: The mitochondrial Hsp70 controls the assembly of the F1FO-ATP synthase
Source: Nat Commun. 2023 Jan 3;14:39. doi: 10.1038/s41467-022-35720-5 (PMC9810599; doi:10.1038/s41467-022-35720-5)
Supplement: Supplementary file 7 — Reporting Summary [file 41467_2022_35720_MOESM7_ESM.pdf]

Corresponding author(s): Thomas Becker

Last updated by author(s): Oct 25, 2022

Reporting Summary

Nature Portfolio wishes to improve the reproducibility of the work that we publish. This form provides structure for consistency and transparency in reporting. For further information on Nature Portfolio policies, see our Editorial Policies and the Editorial Policy Checklist.

Statistics

For all statistical analyses, confirm that the following items are present in the figure legend, table legend, main text, or Methods section.

n/a	Confirmed	
☐	☒	The exact sample size (*n*) for each experimental group/condition, given as a discrete number and unit of measurement
☐	☒	A statement on whether measurements were taken from distinct samples or whether the same sample was measured repeatedly
☐	☒	The statistical test(s) used AND whether they are one- or two-sided<br>*Only common tests should be described solely by name; describe more complex techniques in the Methods section.*
☒	☐	A description of all covariates tested
☒	☐	A description of any assumptions or corrections, such as tests of normality and adjustment for multiple comparisons
☐	☒	A full description of the statistical parameters including central tendency (e.g. means) or other basic estimates (e.g. regression coefficient) AND variation (e.g. standard deviation) or associated estimates of uncertainty (e.g. confidence intervals)
☐	☒	For null hypothesis testing, the test statistic (e.g. F, t, r) with confidence intervals, effect sizes, degrees of freedom and P value noted<br>*Give P values as exact values whenever suitable.*
☒	☐	For Bayesian analysis, information on the choice of priors and Markov chain Monte Carlo settings
☒	☐	For hierarchical and complex designs, identification of the appropriate level for tests and full reporting of outcomes
☒	☐	Estimates of effect sizes (e.g. Cohen's d, Pearson's r), indicating how they were calculated

Our web collection on statistics for biologists contains articles on many of the points above.

Software and code

Policy information about availability of computer code

Data collection	ImageQuant LAS-4000 (GE Healthcare); Amersham Imager 680 (Cytiva); Odyssey DLx Imager (Li-Cor); Typhoon FLA 9000 (GE Healthcare)
Data analysis	Graphpad Prism 9.3.1 (471); Fuji Java 8 software (Wayne Rasband, National Institute of Health, USA); MaxQuant (version 1.6.2.1; https://maxquant.net/maxquant/); Multi Gauge (Fujifilm, V3.2); Image Studio Lite (https://www.licor.com/bio/image-studio-lite/); Adobe Illustrator 2022 (V26.3); Adobe Photoshop 2022 (V23.3.2); Spetronaut 14.7.20 (Biognosys); R Studio 4.1.0 (R Core Team)

For manuscripts utilizing custom algorithms or software that are central to the research but not yet described in published literature, software must be made available to editors and reviewers. We strongly encourage code deposition in a community repository (e.g. GitHub). See the Nature Portfolio guidelines for submitting code & software for further information.

Data

Policy information about availability of data

All manuscripts must include a data availability statement. This statement should provide the following information, where applicable:

- Accession codes, unique identifiers, or web links for publicly available datasets
- A description of any restrictions on data availability
- For clinical datasets or third party data, please ensure that the statement adheres to our policy

The mass spectrometry data have been deposited to the ProteomeXchange Consortium via the PRIDE partner repository and are publicly available under the identifier PXD033024. The authors declare that all data supporting the findings of this study are available within the article or the Supplementary Information. All other data can be obtained by the corresponding authors upon reasonable request. Source data are provided within this paper.

Field-specific reporting

Please select the one below that is the best fit for your research. If you are not sure, read the appropriate sections before making your selection.

☒ Life sciences ☐ Behavioural & social sciences ☐ Ecological, evolutionary & environmental sciences

For a reference copy of the document with all sections, see nature.com/documents/nr-reporting-summary-flat.pdf

Life sciences study design

All studies must disclose on these points even when the disclosure is negative.

Sample size	Sample sizes used for biochemical analysis such affinity purifications, import assays, mass spectrometry, blue native electrophoresis were selected based on previous experiences (e.g. Martensson et al., Nature 2019; Priesnitz et al., Method Cell Biology 2021). Based on these data, the amount of mitochondria or cells for the assays were determined. Independent experiments were performed to confirm the reproducibility of the presented data.
Data exclusions	All relevant data shown. No data were excluded from the analysis.
Replication	For all data shown, the attempts of replication were successful and results are available. The number of replicas of each experiments is specified in the reproducibility, statistics and image processing section.
Randomization	All the clones of yeast strains were selected randomly. For biochemical studies, mitochondria were isolated. The usage of mitochondria for the experiments was not randomized. All samples of one experiment were treated in parallel under the same conditions.
Blinding	Blinding was not performed. The yeast srains had to be validated before they were used for experiments. Blinding is not feasable for biochemical and cell biological assays since all samples were treated in parallel under the same conditions.

Reporting for specific materials, systems and methods

We require information from authors about some types of materials, experimental systems and methods used in many studies. Here, indicate whether each material, system or method listed is relevant to your study. If you are not sure if a list item applies to your research, read the appropriate section before selecting a response.

Materials & experimental systems

n/a	Involved in the study	
☐	☒	Antibodies
☐	☒	Eukaryotic cell lines
☒	☐	Palaeontology and archaeology
☒	☐	Animals and other organisms
☒	☐	Human research participants
☒	☐	Clinical data
☒	☐	Dual use research of concern

Methods

n/a	Involved in the study	
☒	☐	ChIP-seq
☒	☐	Flow cytometry
☒	☐	MRI-based neuroimaging

Antibodies

Antibodies used

Antibodies against proteins from baker´s yeast Saccharomyces cerevisiae were generated in rabbits using peptides from yeast proteins. All antibodies used in this study are listed with detailed information in Supplementary Table 4.
Atp1, 1 : 500, GR5075-3
Atp2, 1 : 1000, GR863-4
Atp3, 1 : 250, GR1671-4
Atp4, 1 : 250, GR1970-4
Atp5, 1 : 250, GR1546-4
Atp11, 1 : 200, GR5152-2
Atp12, 1 : 200, GR5155-2
Atp19, 1 : 250, GR1961-3
Atp20, 1 : 1000, GR1516-4
Ina17 1 : 500, GR3340-2
mtHsp70, 1 : 1000, GR1830-3
Hsp60, 1 : 1000, GR170-2
Cox2, 1 : 250, GR1948-4
Cox4, 1 : 1000, GR578-5
Cox9, 1 : 500, GR3611-3
Sdh1, 1 : 250, GR1848-5
Rip1, 1 : 1500, GR543-4

Mge1, 1 : 500, GR1837-2
Mdh1, 1 : 1000, GR1089-4
Tom40, 1 : 1000, GR168-5
Tom70, 1 : 500, GR657-3
Tim44, 1 : 250, GR1835-4
Tim50, 1 : 250, GR3881-3
Tim17, 1 : 500, GR1844-6
Tim23, 1 : 500, GR3878-4
Pam16, 1 : 250, GR3121-4
Pam18, 1 : 250, GR751-2
Mdj1, 1 : 250, GR121-7
Aco1, 1 : 1000, GR945-3
Pim1, 1 : 250, GR626-5

References:
Rampelt, H. et al. Dual role of Mic10 in mitochondrial cristae organization and ATP synthase-linked metabolic adaptation and respiratory growth. Cell Rep. 38,110290 (2022)
Böttinger, L. et al. A complex of Cox4 and mitochondrial Hsp70 plays an important role in the assembly of the cytochrome c oxidase. Mol. Biol. Cell 24, 2609-2619 (2013).
Lytovchenko, O. et al. The INA complex facilitates assembly of the peripheral stalk of the mitochondrial F1FO-ATP synthase. EMBO J. 33, 1624-38 (2014).
Böttinger, L. et al. Mitochondrial heat shock protein (Hsp) 70 and Hsp10 cooperate in the formation of Hsp60 complexes. J. Biol. Chem. 290, 11611-11622 (2015).
Mårtensson, C.U. et al. Mitochondrial protein translocation-associated degradation. Nature 569, 679-683 (2019)
Priesnitz, C. et al. Coupling to Pam16 differentially controls the dual role of Pam18 in protein import and respiratory chain formation. Cell Rep. 39, 110619 (2022).
Bender, T., Leidhold, C., Ruppert, T., Franken, S. & Voos, W. The role of protein quality control in mitochondrial protein homeostasis under oxidative stress. Proteomics 10, 1426-1443 (2010)

Secondary antibodies were obtained from Dianova (HRP-conjugated goat anti-rabbit, Cart. 111-035-003) or Li-Cor (goat anti-mouse IgG, IRDye 800CW, Cat. 926-32210; goat anti-rabbit IgG, IRDye 800CW, Cat. 926-32211; goat anit-mouse IgG, IRDye 680RD, Cat. 926-68070, goat anti-rabbit IgG, IRDye 680RD, Cat. 926-68071). Secondary antibodies were used at a concentration of 1:5,000 (HRP) or 1:10,000 (IRDye).

Validation

All the antisera in use have been tested by separating proteins from mitochondria or cell extracts on SDS-PAGE followed by immunoblotting. Mitochondria and cell extracts were prepared from WT and the corresponding deletion strain or yeast strain expressing a tagged version of the protein. Absence or size shift of the band in samples from the mutant strains shows specificity of the antisera.

Eukaryotic cell lines

Policy information about cell lines

Cell line source(s)

The model organism of this study is baker's yeast Saccharomyces cerevisiae. All yeast strains used in this study are listed with detailed information in Supplementary Table 4.
The following yeast strains were purchase from Euroscarf (http://www.euroscarf.de/search.php?name=Order):
BY4741 (WT), atp3Δ, atp4Δ, atp5Δ, atp7Δ, atp14Δ, ina17Δ, ina22Δ, pim1Δ, yta12Δ

Yeast trains YPH499 (WT), mtHsp70His, ssc1-62, Tim17His have been described:
Sikorski, R.S. & Hieter, P. A system of shuttle vectors and yeast host strains designed for efficient manipulation of DNA in Saccharomyces cerevisiae. Genetics 122, 19-27 (1989).

Böttinger, L. et al. A complex of Cox4 and mitochondrial Hsp70 plays an important role in the assembly of the cytochrome c oxidase. Mol Biol Cell 24, 2609-19 (2013).

Priesnitz, C. et al. Coupling to Pam16 differentially controls the dual role of Pam18 in protein import and respiratory chain formation. Cell Rep. 39, 110619 (2022).

Yeast strain BY4741 ρ0 was generated via ethidium bromide treatment and the lack of mitochondrial DNA was confirmed by PCR (data not shown).

The following yeast strains were newly generated for this study:
ρ0 mtHsp70His
Atp1His
Atp11His
Atp12His
Ina17His
Ina22His
ssc1-62 Atp11His
ssc1-62 Atp12His
ssc1-62 Ina17His
ssc1-62 Ina22His
Ssc1 WT ina17Δ
Ssc1 WT ina22Δ
Ssc1 WT fmc1Δ

ssc1-62 ina17Δ
ssc1-62 ina22Δ
ssc1-62 fmc1Δ
Ssc1 WT + pRS415
Ssc1 WT + pRS415-GAL1-ATP5
Ssc1 WT + pRS415-GAL1-ATP5 G183A
Ssc1 WT + pRS415-GAL1-ATP5HA
Ssc1 WT + pRS415-GAL1-ATP5HA G183A
ssc1-62 + pRS415
ssc1-62 + pRS415-GAL1-ATP5
ssc1-62 + pRS415-GAL1-ATP5 G183A
ssc1-62 + pRS415-GAL1-ATP5HA
ssc1-62 + pRS415-GAL1-ATP5HA G183A
pim1Δ + pRS415-GAL1-ATP5HA
pim1Δ + pRS415-GAL1-ATP5HA G183A
yta12Δ + pRS415-GAL1-ATP5HA
yta12Δ + pRS415-GAL1-ATP5HA G183A

Authentication

Yeast strains in use were cultured on the required medium of selection. Deletion or tagging or overexpression of proteins of interests shows absent, shifted or increased signal respectively on western blot with corresponding antisera.

Mycoplasma contamination

Mycoplasma contamination is not relevant for yeast cells was therefore not analyzed.

Commonly misidentified lines
(See ICLAC register)

No commonly misidentified cell lines were used in this study.
