# Supplementary material for: The mitochondrial Hsp70 controls the assembly of the F1FO-ATP synthase
Source: Nat Commun. 2023 Jan 3;14:39. doi: 10.1038/s41467-022-35720-5 (PMC9810599; doi:10.1038/s41467-022-35720-5)
Supplement: Supplementary file 8 — Source Data [file 41467_2022_35720_MOESM8_ESM.zip › Source Data_uncropped blots.pdf]

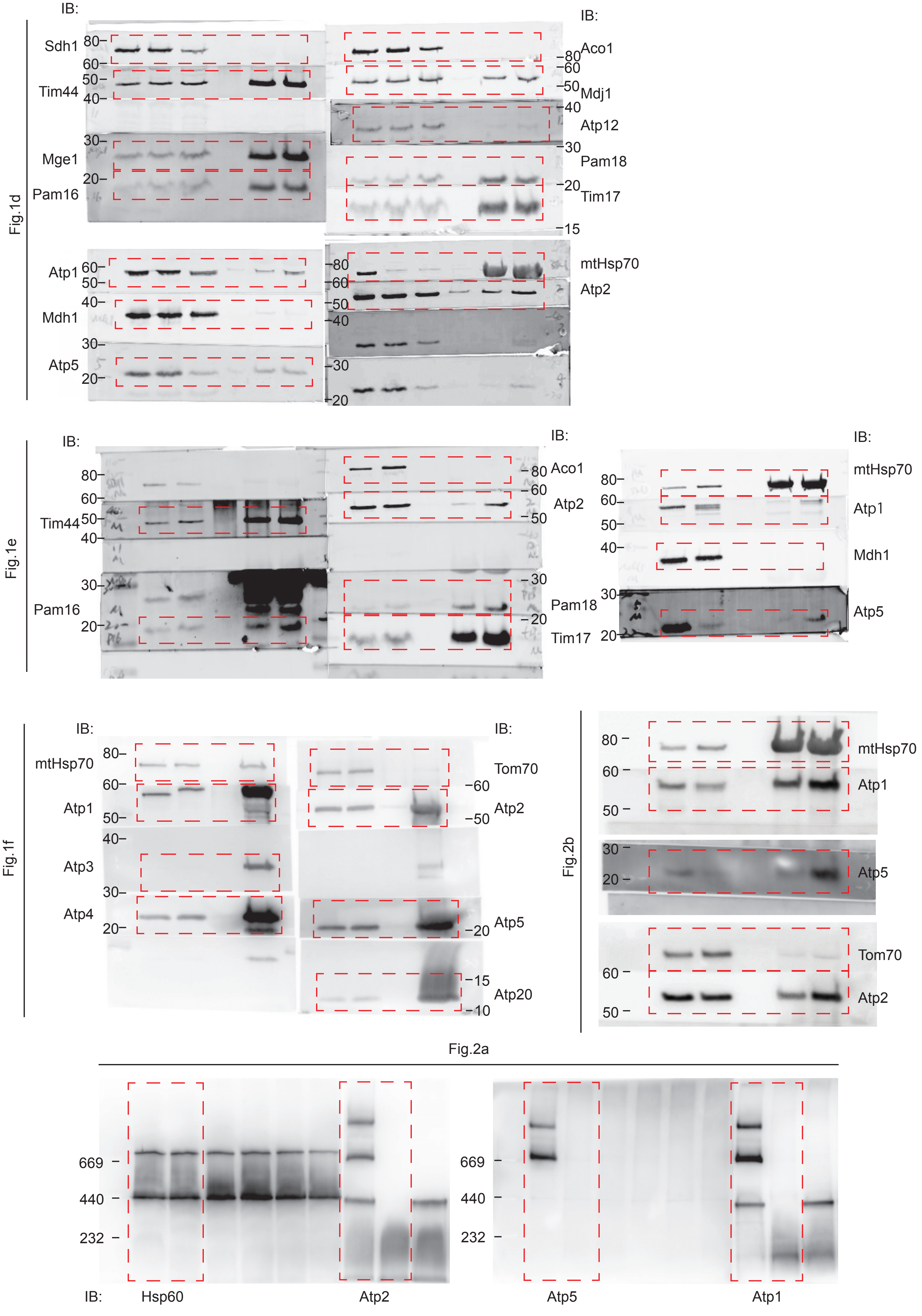

Fig.1d
IB:
Sdh1
Tim44
Mge1
Pam16
Aco1
Mdj1
Atp12
Pam18
Tim17
Atp1
Mdh1
Atp5
mtHsp70
Atp2
80
60
50
40
30
20
15
Fig.1e
IB:
Tim44
Pam16
Aco1
Atp2
Pam18
Tim17
mtHsp70
Atp1
Mdh1
Atp5
Fig.1f
IB:
mtHsp70
Atp1
Atp3
Atp4
Tom70
Atp2
Atp5
Atp20
10
Fig.2b
mtHsp70
Atp1
Atp5
Tom70
Atp2
Fig.2a
669
440
232
IB:
Hsp60
Atp2
Atp5
Atp1

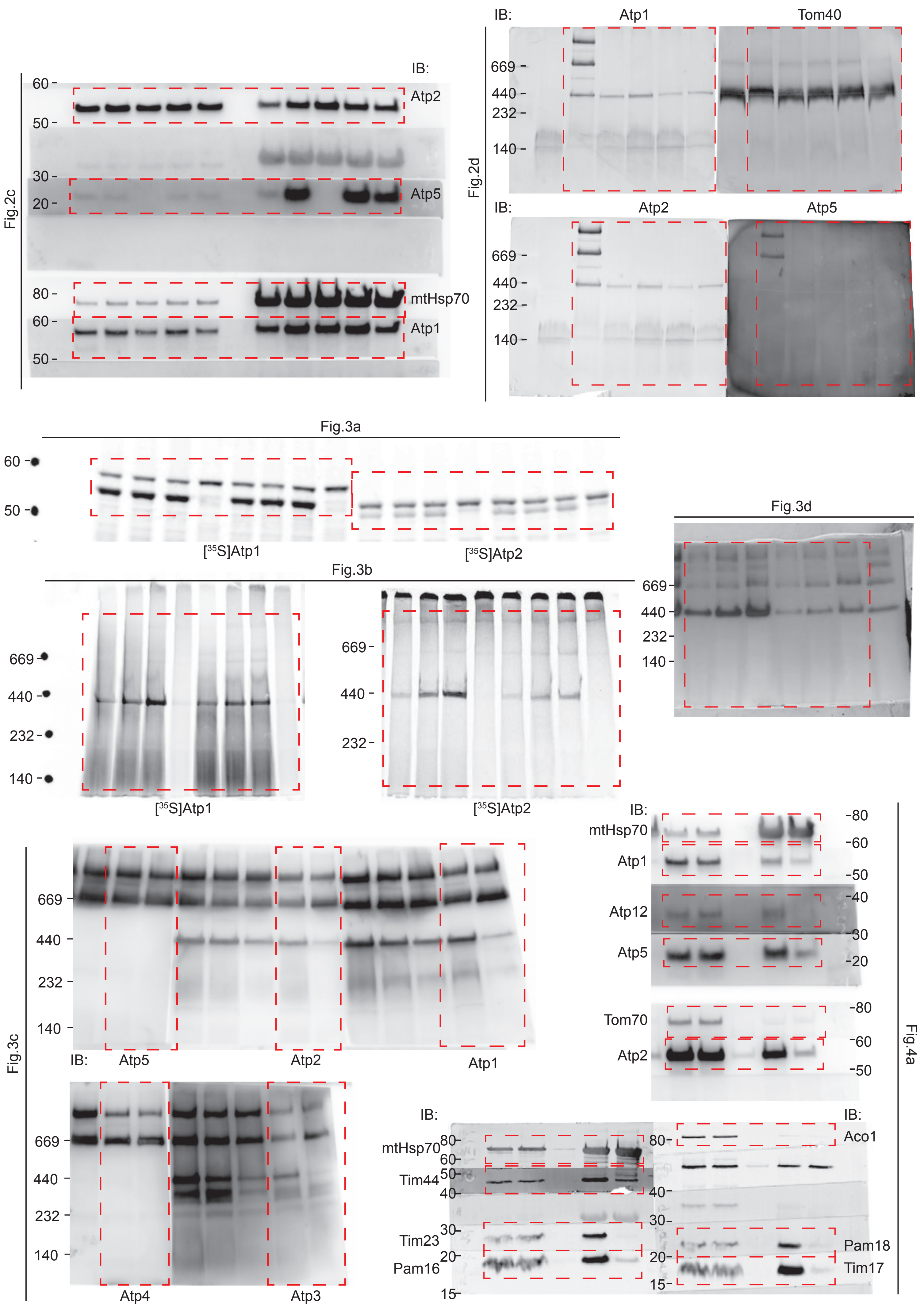
Fig.2c
IB:
Atp2
Atp5
mtHsp70
Atp1
60
50
30
20
80
60
50
Fig.2d
IB:
Atp1
Tom40
669
440
232
140
Atp2
Atp5
Fig.3a
60
50
[35S]Atp1
[35S]Atp2
Fig.3b
669
440
232
140
[35S]Atp1
[35S]Atp2
Fig.3d
Fig.3c
IB:
Atp5
Atp2
Atp1
Atp4
Atp3
Fig.4a
IB:
mtHsp70
Atp1
Atp12
Atp5
Tom70
Atp2
80
60
50
40
30
20
Tim44
Tim23
Pam16
Aco1
Pam18
Tim17
15

Fig.4b

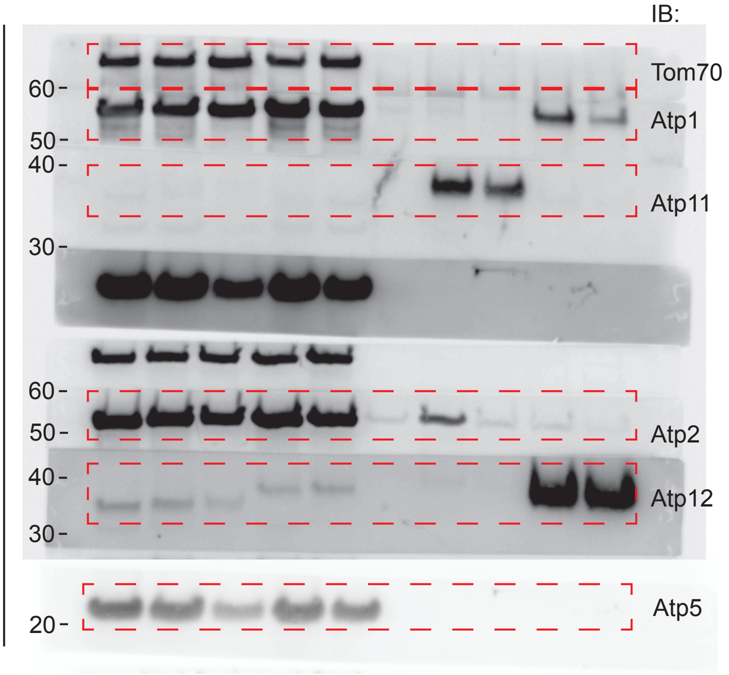

Fig.4c

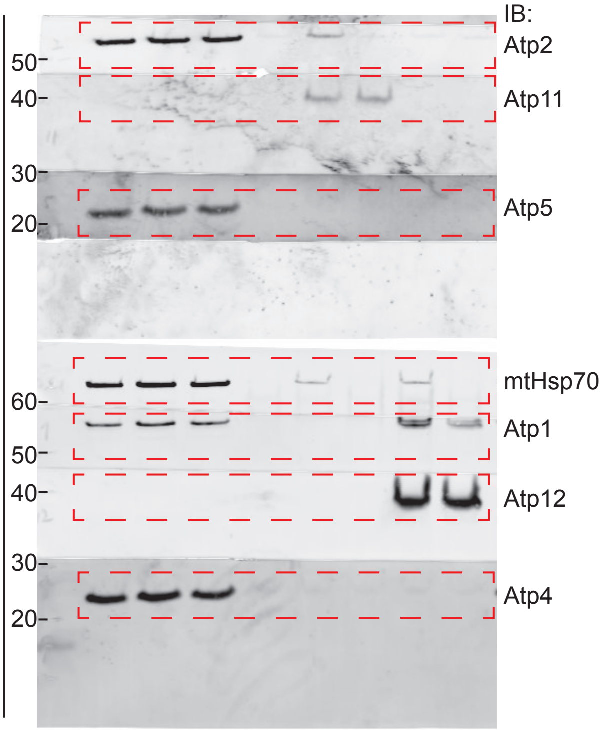

Fig.4e

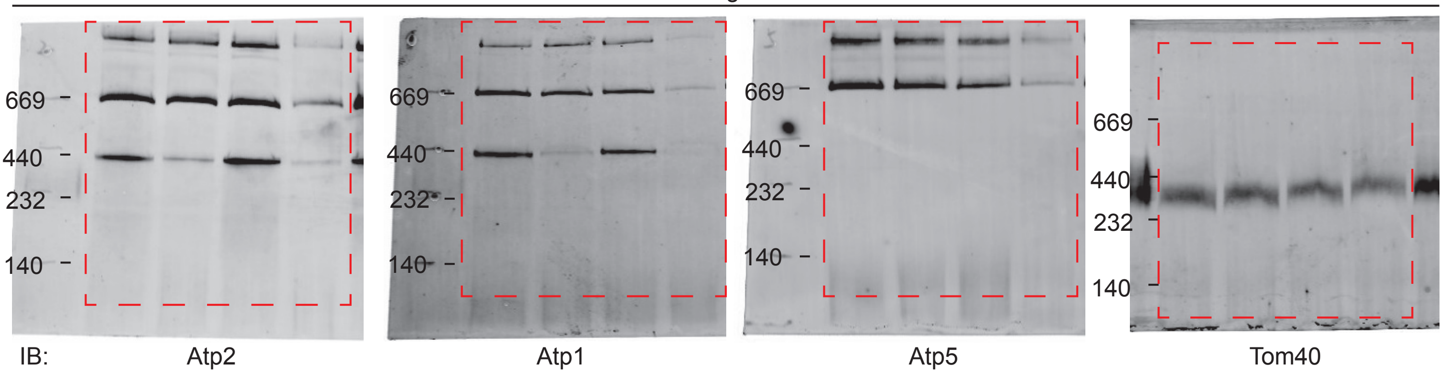

Fig.5a

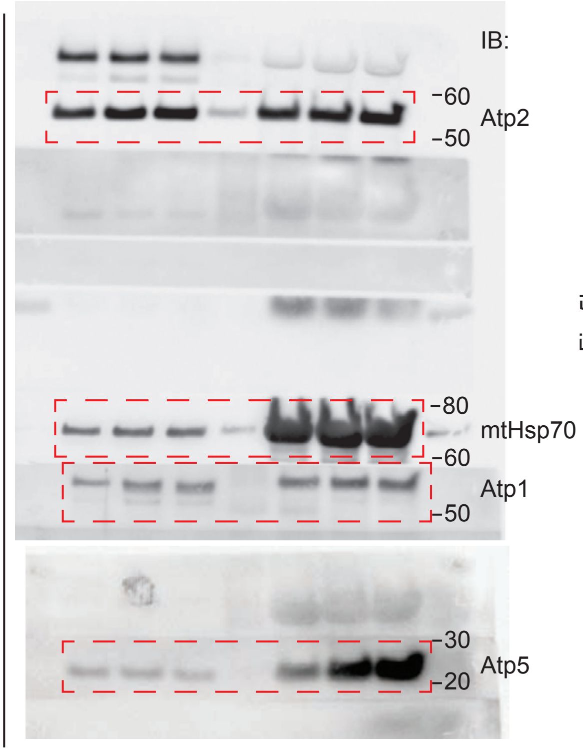

Fig.5b

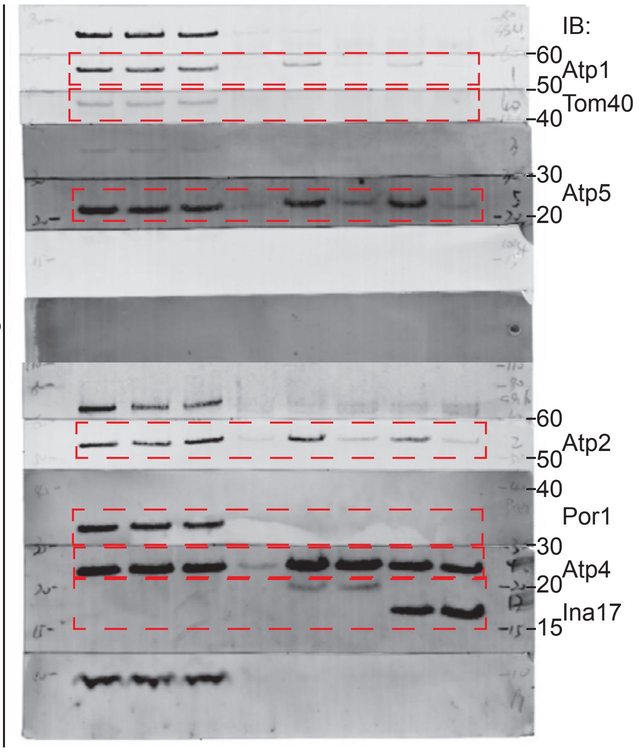

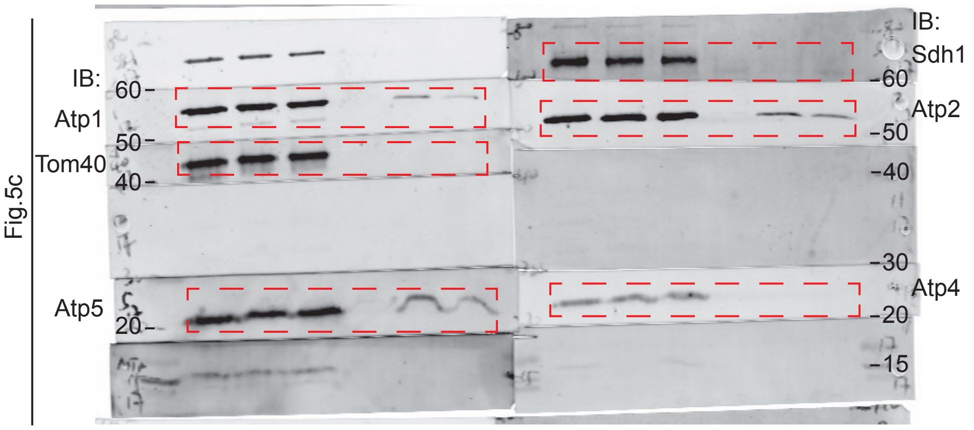

Fig.5e

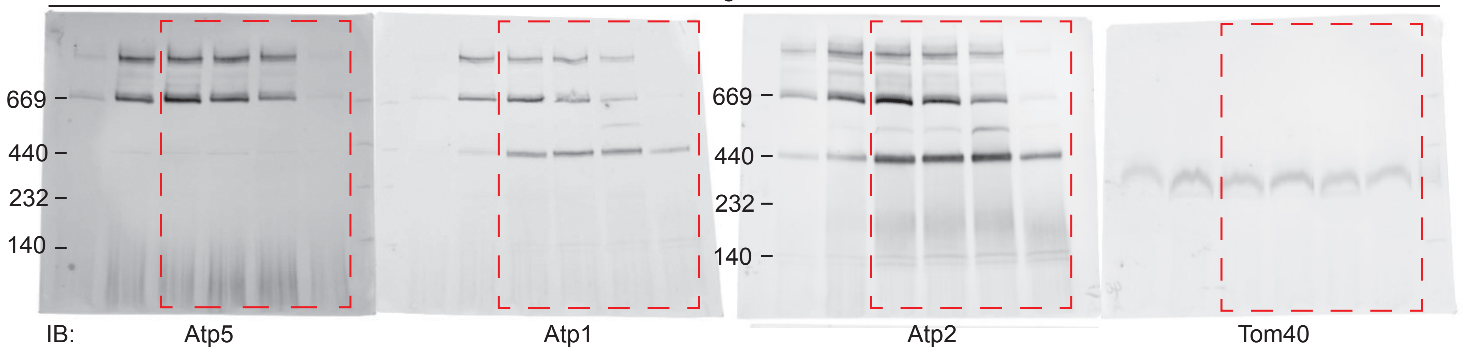

Fig.6a

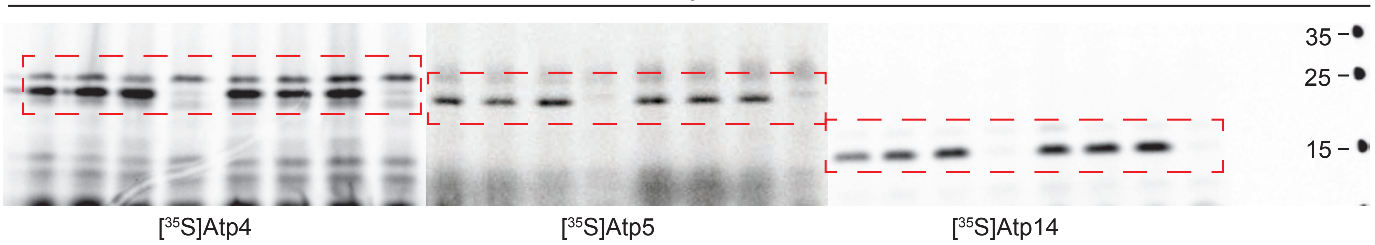

Fig.6b

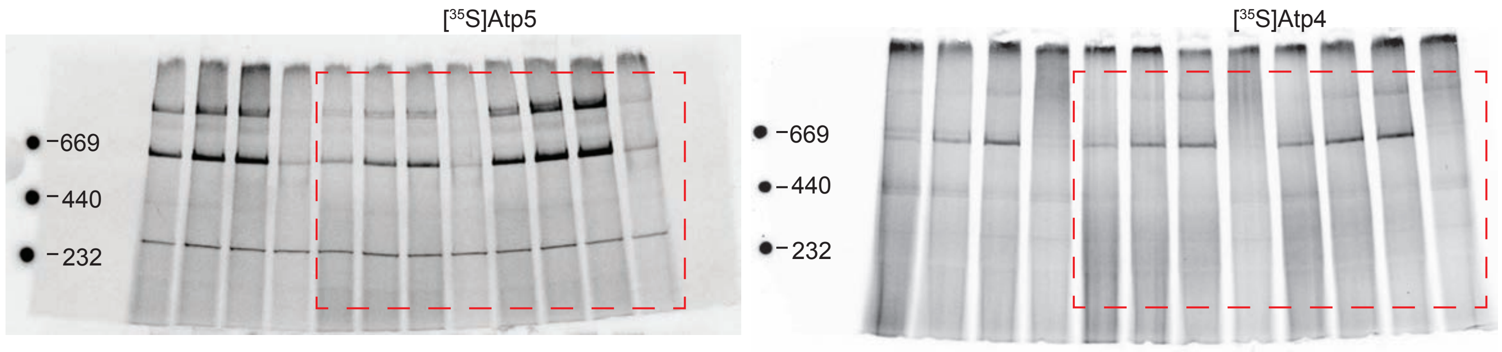

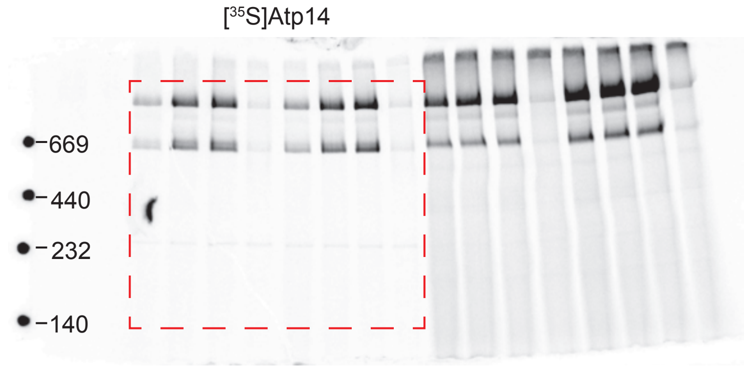

Fig.6c

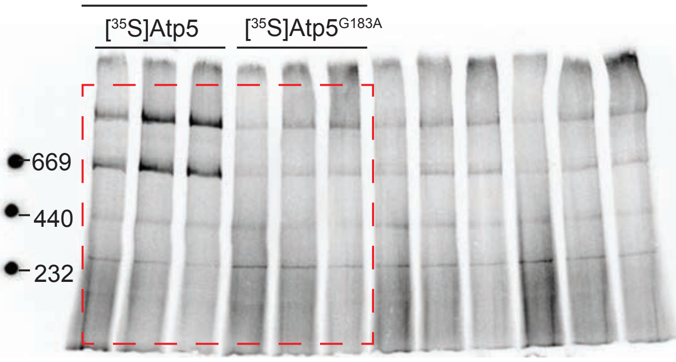

Fig.6d

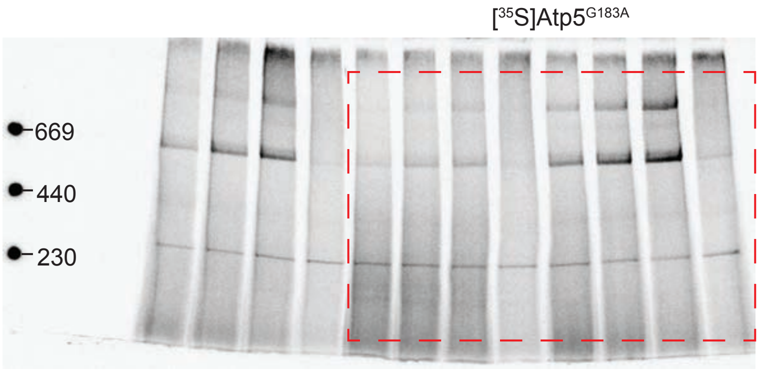

Fig.7d

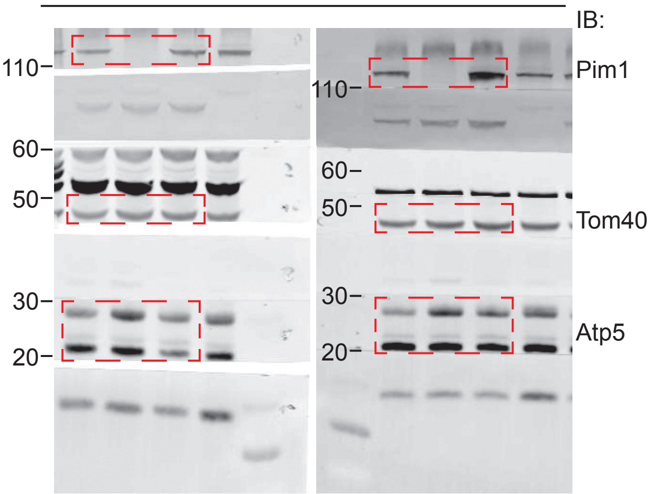

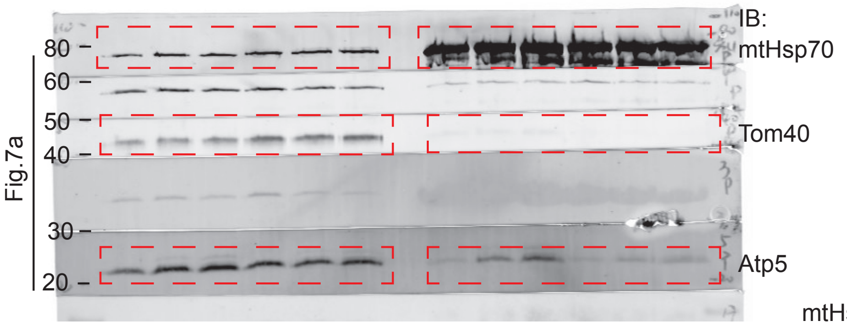

SFig.1b

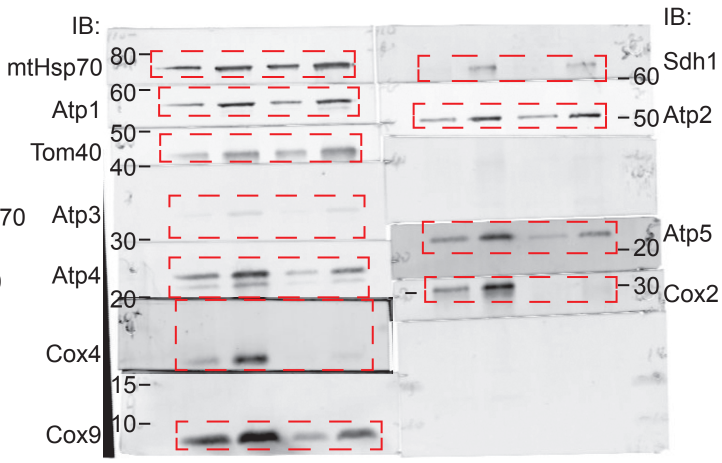

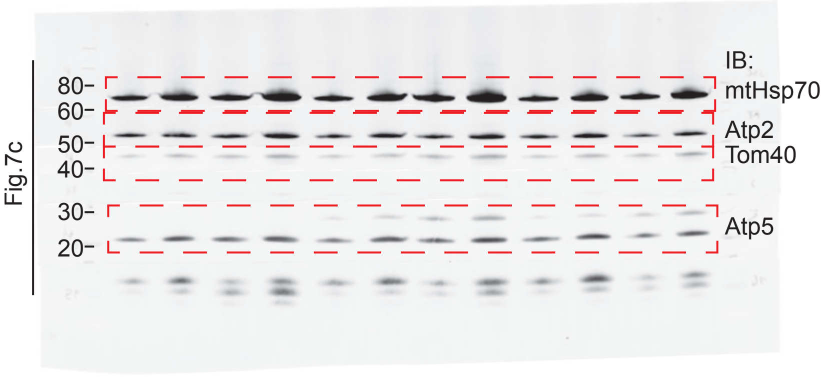

SFig.1c

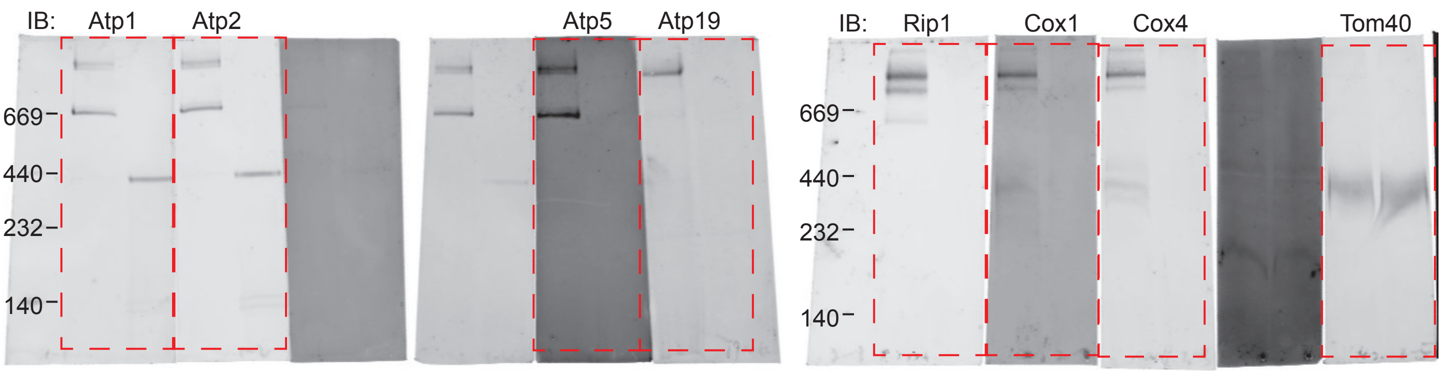

SFig.1d

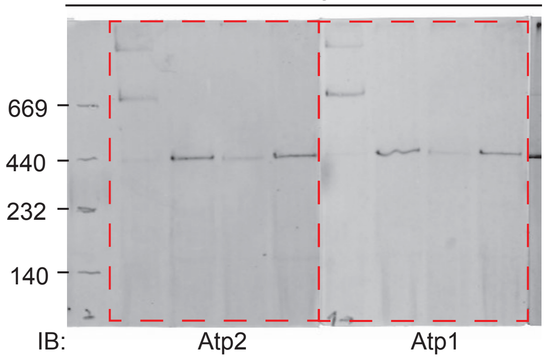

SFig.1e

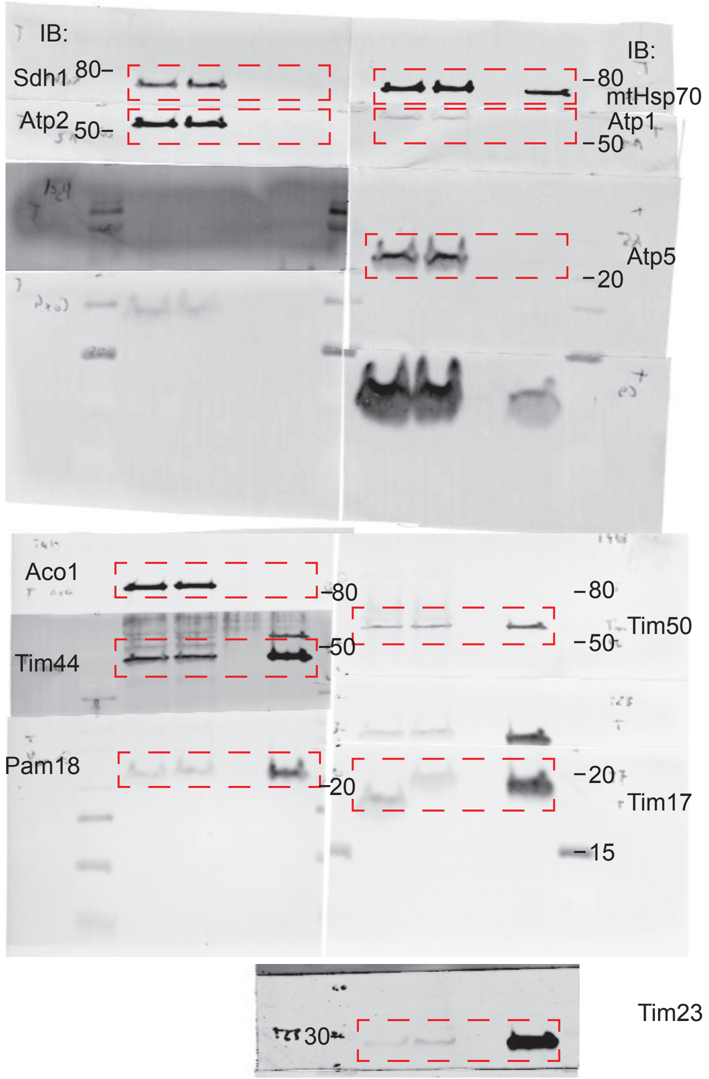

SFig.1f

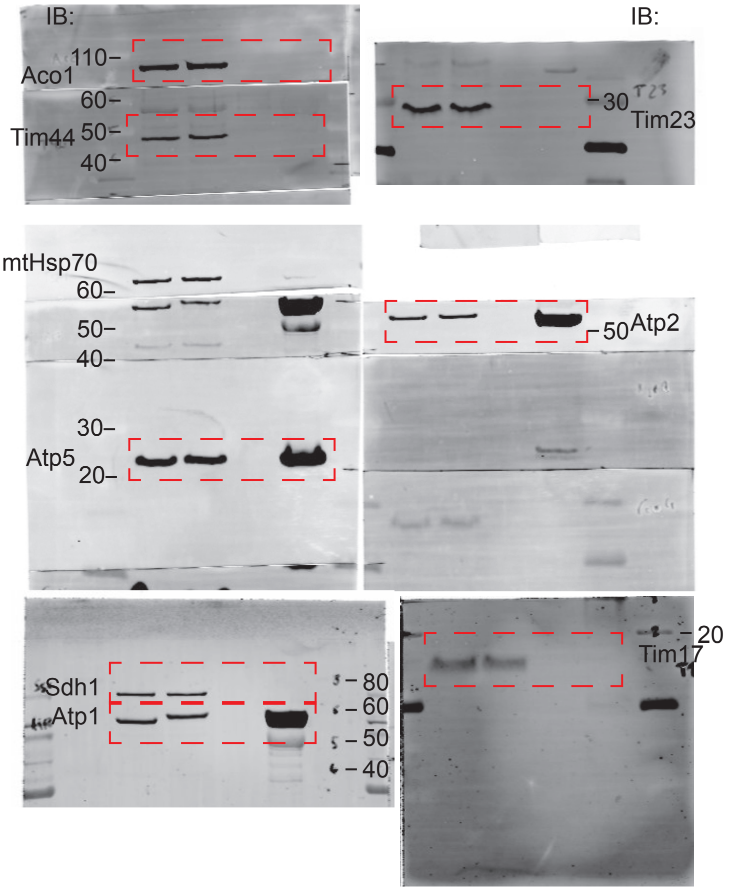

SFig.2a

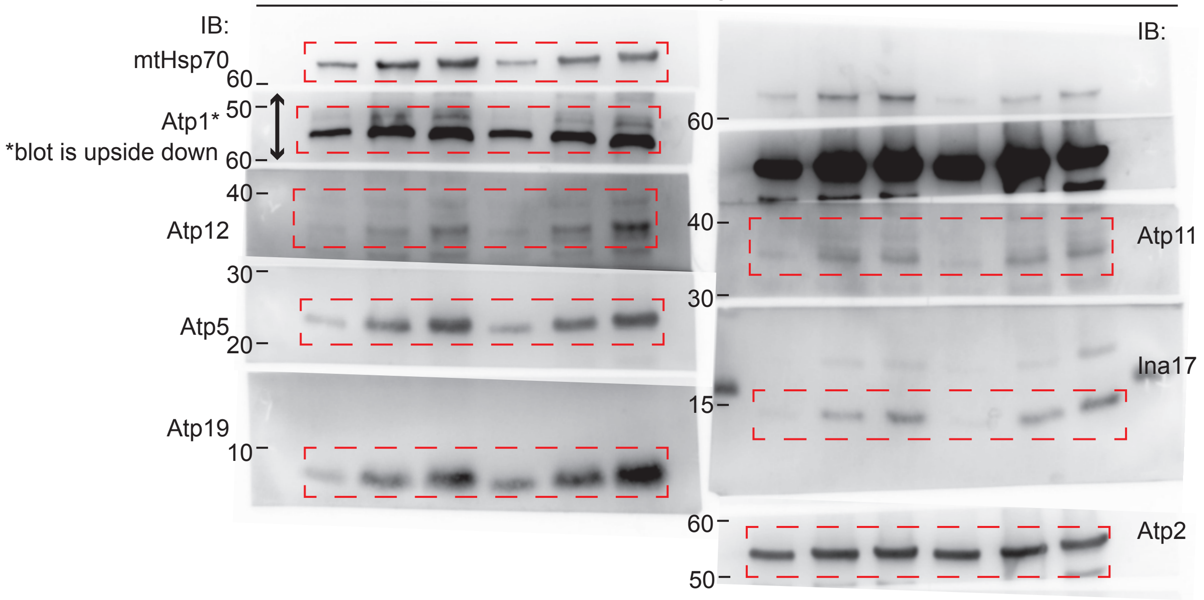

SFig.2b

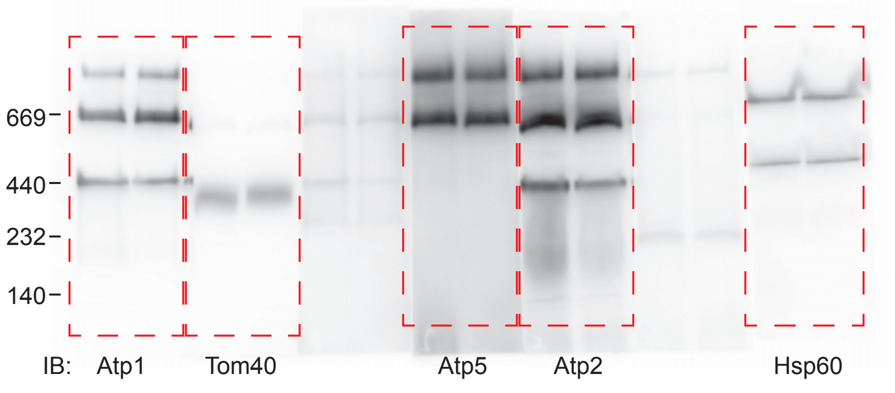

SFig.3a

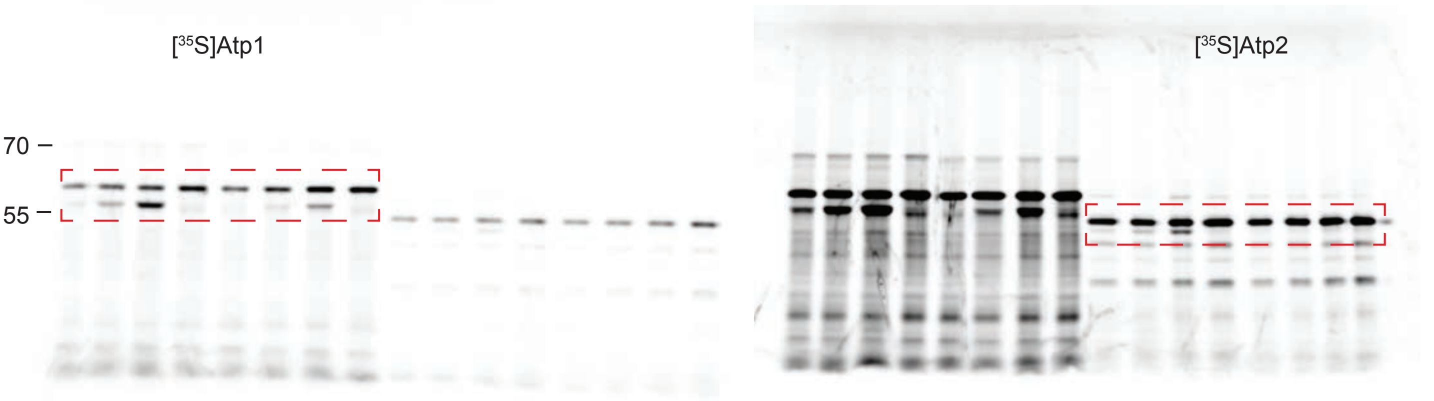

SFig.3b

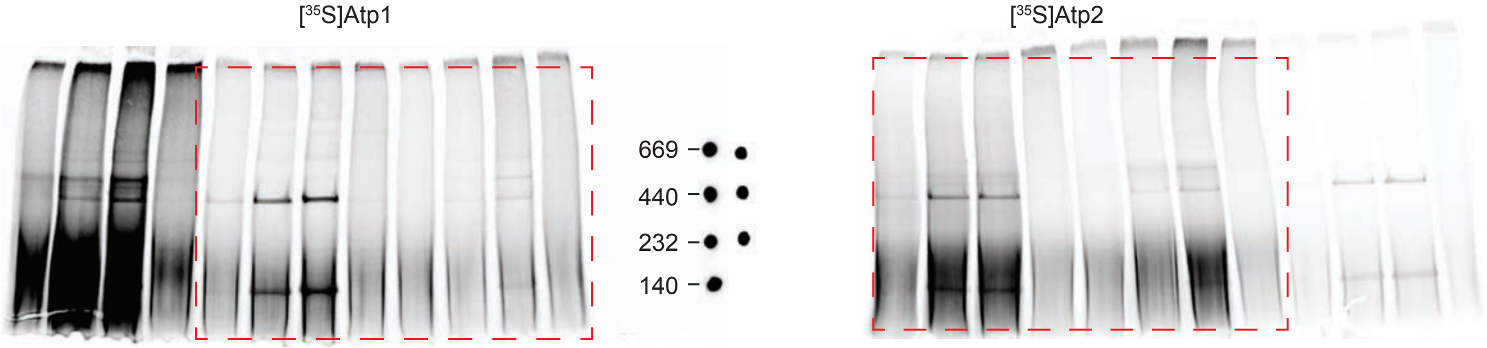

SFig.4b

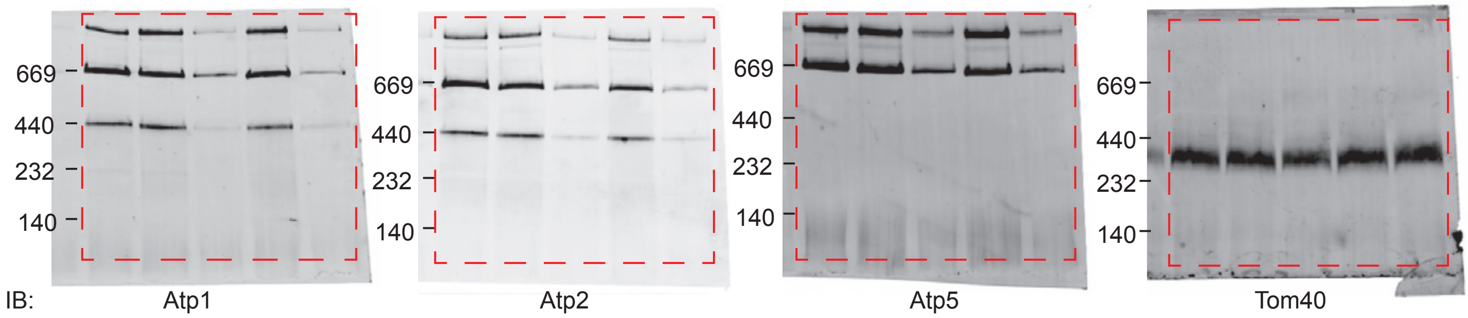

SFig.5a

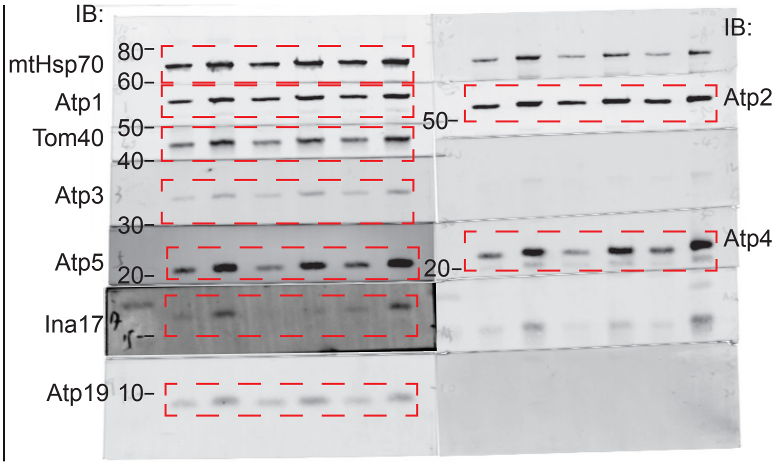

SFig.5b

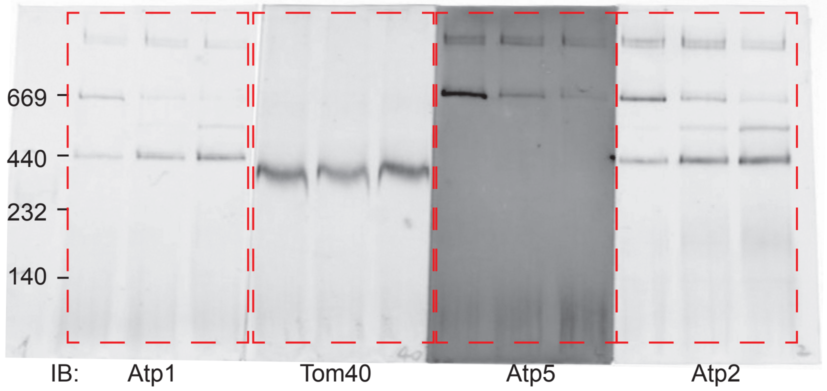

SFig.5c

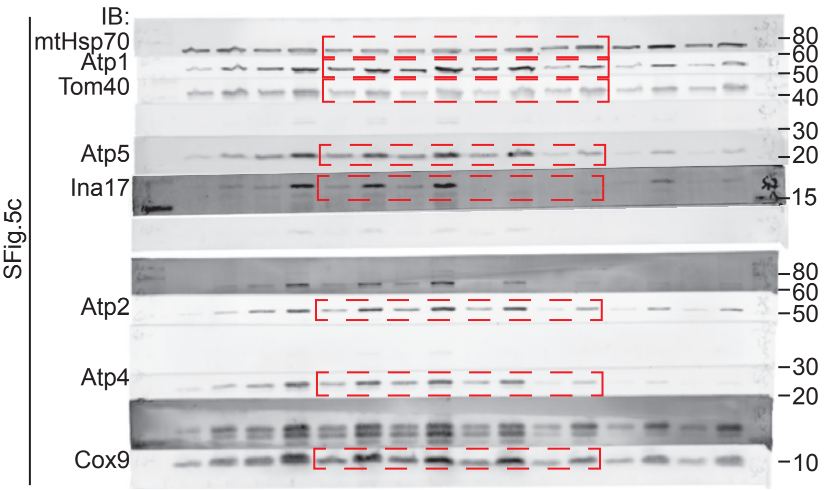

SFig.6a

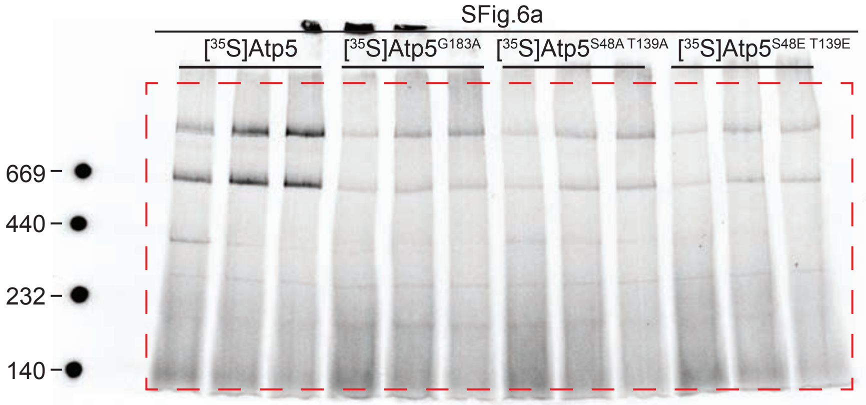

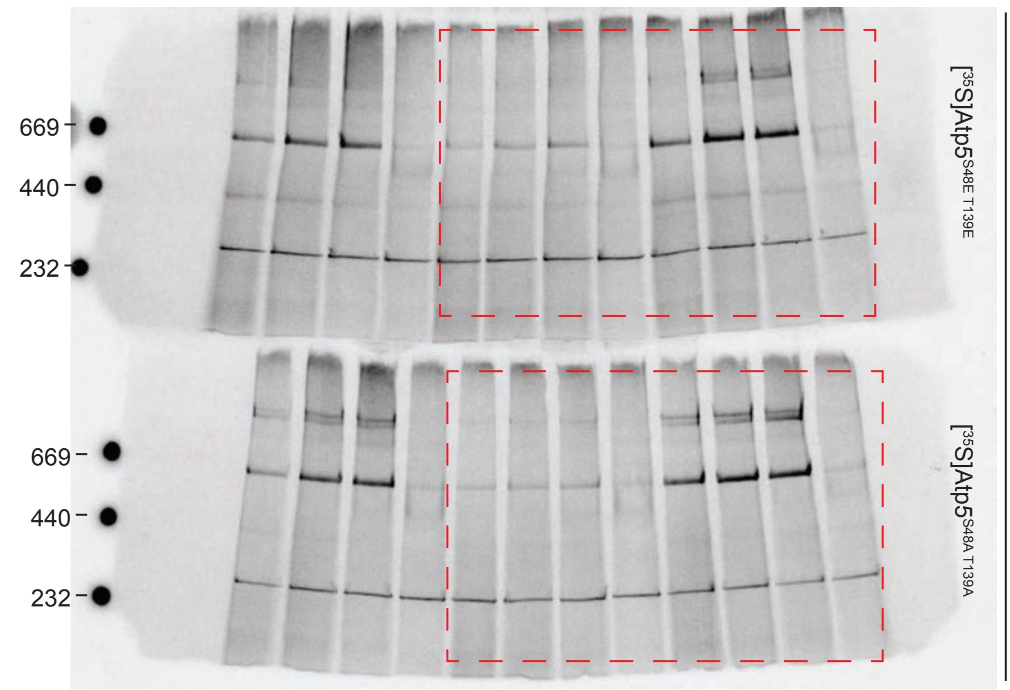

SFig.6b
